# The signature of fine scale local adaptation in Atlantic salmon revealed from common garden experiments in nature

**DOI:** 10.1111/eva.12299

**Published:** 2015-09-11

**Authors:** Ciar L O'Toole, Thomas E Reed, Deborah Bailie, Caroline Bradley, Deirdre Cotter, Jamie Coughlan, Tom Cross, Eileen Dillane, Sarah McEvoy, Niall Ó Maoiléidigh, Paulo Prodöhl, Ger Rogan, Philip McGinnity

**Affiliations:** 1 School of Biological, Earth & Environmental Sciences, University College CorkCork, Ireland; 2 Institute for Global Food Security, School of Biological Sciences, Medical Biology Centre, Queen's UniversityBelfast, Northern Ireland; 3 Marine Institute, FurnaceNewport, Co. Mayo, Ireland

**Keywords:** adaptive peak, anadromous, common garden, gene flow, heterosis, outbreeding depression, spatial scale

## Abstract

Understanding the extent, scale and genetic basis of local adaptation (LA) is important for conservation and management. Its relevance in salmonids at microgeographic scales, where dispersal (and hence potential gene flow) can be substantial, has however been questioned. Here, we compare the fitness of communally reared offspring of local and foreign Atlantic salmon *Salmo salar* from adjacent Irish rivers and reciprocal F_1_ hybrid crosses between them, in the wild ‘home’ environment of the local population. Experimental groups did not differ in wild smolt output but a catastrophic flood event may have limited our ability to detect freshwater performance differences, which were evident in a previous study. Foreign parr exhibited higher, and hybrids intermediate, emigration rates from the natal stream relative to local parr, consistent with genetically based behavioural differences. Adult return rates were lower for the foreign compared to the local group. Overall lifetime success of foreigners and hybrids relative to locals was estimated at 31% and 40% (mean of both hybrid groups), respectively. The results imply a genetic basis to fitness differences among populations separated by only 50 km, driven largely by variation in smolt to adult return rates. Hence even if supplementary stocking programs obtain broodstock from neighbouring rivers, the risk of extrinsic outbreeding depression may be high.

## Introduction

When populations of the same species are fully or partially reproductively isolated, for example due to constraints on dispersal and/or effective gene flow reinforced by natal philopatry, they are expected to evolve along independent trajectories. If selective pressures vary across space, then microevolutionary responses may drive adaptive divergence among populations, i.e. local adaptation (LA; Kawecki and Ebert 2004). Even in the absence of spatial variation in selection, locally co-adapted gene complexes may evolve in isolated breeding populations as mutations arise at random and are selected for their average effects in different genetic backgrounds (Lynch and Walsh 1998). A major issue in applied evolutionary biology thus concerns the mixing of divergent gene pools, as occurs for example when nonlocal plant or animal material is used in ecological restoration programs (Hufford and Mazer 2003; Broadhurst et al. 2008; Endler et al. 2010; Weeks et al. 2011), or nonlocal broodstock or broodstock adapted to hatchery environments are used for supportive fish stocking (Allendorf and Waples 1996; Araki et al. 2008). In such situations, interbreeding between ‘local’ and ‘foreign’ genotypes may result in genetic introgression of nonlocal alleles, which can erode pre-existing genetic structure and lead to loss of fitness via extrinsic outbreeding depression (loss of LA in hybrid individuals exhibiting intermediate trait values, which are suboptimal in the environments of both parent populations) or intrinsic outbreeding depression (reduced positive epistasis or increased negative epistasis due to breakdown of co-adapted gene complexes) (Lynch and Walsh 1998). The goal of the current study was to test for reduced fitness of nonlocal genotypes and experimentally created hybrid (i.e. one nonlocal parent, one local) genotypes in the ‘home’ environment of a local population of Atlantic salmon, *Salmo salar*, an excellent model species in which to examine the potential consequences of intra-specific hybridization and the implications for evolutionary conservation and management.

Salmonid fishes are a group with a long history of study of intraspecific genetic divergence (Ricker 1972; Taylor 1991; Adkison 1995; Garcia de Leaniz et al. 2007; Fraser et al. 2011). Salmonids occupy an array of different habitats from temperate to Arctic regions and experience substantial environmental heterogeneity at both macro-geographic (e.g. different latitudes) and micro-geographic (e.g. adjacent catchments, or different tributaries within the same catchment) scales. Strong natal homing promotes reproductive isolation (Quinn 2005) and hence potentially (semi-)independent evolutionary trajectories among sub-populations spawning in distinct areas or habitats (Allendorf and Waples 1996; Hansen et al. 2002). Understanding ecological and genetic processes driving adaptive population divergence in salmonids has manifold practical implications, including informing the delineation of sub-specific units for conservation and management purposes (Waples 1991; Fraser and Bernatchez 2001), assessment of demographic and genetic risks of interbreeding between wild and farmed salmon (Fleming and Einum 1997; McGinnity et al. 1997, 2003; Hindar et al. 2006; Fraser et al. 2008; Hutchings and Fraser 2008) or stocking programmes (reviewed by Araki et al. 2008), choosing appropriate (e.g. disease resistant, fast growth rate) broodstock in aquaculture operations (Taylor 1991; Myers et al. 2001) and predicting the success of intentional translocations or invasive species in foreign habitats (Westley et al. 2012). Moreover, adaptive differences among populations are also thought to be important both for the resilience and productivity of salmonid stock complexes (Hilborn et al. 2003; Greene et al. 2010; Carlson et al. 2011; Moore et al. 2014). For example, diversity in life histories, phenologies and climate responses (thought to be underpinned by LA) among populations of sockeye salmon (*Onchorynchus nerka*) in Bristol Bay, Alaska results in asynchronous dynamics such that population numbers are more stable at the aggregate level than within single populations (Hilborn et al. 2003; Rogers and Schindler 2008). This accrues benefits for commercial fisheries and mobile consumer species (Schindler et al. 2010, 2013; Ruff et al. 2011).

Although the importance of LA has become an accepted paradigm in salmonid biology, its importance at smaller geographic scales remains relatively understudied (Fraser et al. 2011; but see Westley et al. 2012). Furthermore, modelling work (Adkison 1995) suggests that random genetic differentiation of populations or genetic homogeneity (despite phenotypic heterogeneity) might be just as likely under a broad range of realistic conditions including, for instance weak or inconsistent selection differentials, low and variable population sizes, high straying rates and founder effects related to extinction-recolonization dynamics. In a recent meta-analysis, Fraser et al. (2011) emphasised that while there is evidence for LA in salmonids at a range of spatial scales, its frequency and magnitude is generally greater at larger geographic scales (>100–200 km). Key to interpreting the spatial scale of adaptation in any species, however, is the species’ dispersal capability (Moore et al. 2013; Richardson et al. 2014). Although salmon are renowned for natal philopatry, straying among rivers does occur and genetic evidence suggests that dispersal among distant regions is not infrequent (Dionne et al. 2008). At the same time, however, pronounced genetic differentiation is often found over small spatial scales (e.g. <50 km) in salmonids, and population structure may be shaped as much by genetic drift and/or ‘isolation by adaptation’ as by straying (Hendry et al. 2000; Lin et al. 2008; Bradbury et al. 2013; Bond et al. 2014; Larson et al. 2014). Hence, a tension likely exists in salmonids at microgeographic scales (Richardson et al. 2014) between the diversifying effects of spatially variable selection and the homogenizing effects of gene flow; thus LA may be a less certain outcome at these scales. In line with this, Fraser et al. (2011) documented considerable variability in the extent of LA at scales of ≤100–200 km and called for more studies at finer geographic resolutions.

While a range of approaches exists for detecting evidence consistent with LA in salmonids (reviewed by Fraser et al. 2011), the ‘gold standards’ remain common-garden field experiments and reciprocal transplants. Ideally, full reciprocals should be carried out; if local individuals have higher fitness than foreigners in the home habitat of the local population and individuals perform better in their home habitat than in a foreign habitat, LA is strongly implicated. Of the two diagnostics, the ‘local versus foreign’ criterion is considered the most reliable, given that ‘home versus away’ comparisons may confound LA with intrinsic habitat differences (Kawecki and Ebert 2004; Fraser et al. 2011; Westley et al. 2012).The inclusion of hybrid crosses between source populations can also help reveal underlying interactions between gene flow and natural selection (Hatfield and Schluter 1999; Gilk et al. 2004; Kawecki and Ebert 2004). In particular, demonstrating that hybrids exhibit intermediate fitness (or trait values linked to fitness) to superior natives and inferior non-natives under communal conditions provides compelling evidence for genetically based LA (Hines et al. 2004). Intrinsic outbreeding depression would also be indicated if the mean fitness of hybrids is reduced below additive expectation (i.e. the mean of the two parents in the test environment). Alternatively, heterosis (‘hybrid vigour’) could result, where the fitness of hybrids is higher than either pure type as a result of the masking of deleterious recessive alleles, which are more likely to have accumulated when original parental population sizes were small (Lynch and Walsh 1998). Here, we report on a common garden field-experiment where the freshwater survival, marine performance and fitness-related phenotypes of wild Atlantic salmon (*Salmo salar*) from two adjacent catchments in the west of Ireland were compared in the home environment of one of them. A full reciprocal was unfortunately not possible, but as outlined above, demonstrating local versus foreign advantage in a single environment still provides powerful evidence that is consistent with LA. A previous common garden experiment (McGinnity et al. 2004) involving these same two populations found that egg-to-smolt survival and smolt-to-returning adult survival for non-native parents was 65% and 21% that of native parents, respectively. These results involving wild salmon from adjacent catchments were dramatic and suggested that LA might operate at a very small geographic scale, particularly in relation to the marine phase of the lifecycle. We wished to test whether such results were temporally stable. Also, hybrid crosses were not included in the previous study, hence, by including them here, we wanted to test for selection against intermediate forms and nonadditive genetic effects on fitness components (e.g. heterosis), thus further examining the processes of LA and outbreeding depression. Therefore, in the present study, first generation (F1) hybrids between native and non-native Atlantic salmon were created by artificial fertilization and their traits and performance at different life stages assessed relative to pure natives and pure non-natives under communal conditions. The pure ‘foreign’ group in our experiment emulates a scenario where nonlocal broodstock are used in fish hatchery operations and the resulting offspring are then stocked into the local watershed (i.e. the ‘home’ environment for the local population), which was once common practice in salmonids and is still carried out to some extent. Furthermore, by including hybrid crosses between a ‘local’ and a ‘foreign’ population, this emulates a scenario where immigrants from foreign populations interbreed with locals. Such ‘straying’ occurs naturally to some extent in anadromous salmonids but straying rates may also be higher among hatchery-produced fish (Quinn 2005), and similarly unnatural hybridization between divergent gene pools can occur when farm salmon escapees spawn in the wild (Glover et al. 2012). If LA is important, we hypothesised that locals should have higher stage-specific survival rates and higher overall fitness than foreigners in the home environment for the locals, while hybrids should have intermediate fitness (Kawecki and Ebert 2004). On the basis of previous findings in salmonids generally (reviewed by Fraser et al. 2011) and this population specifically (McGinnity et al. 2004), we expected to find these signatures of LA/outbreeding depression during both the freshwater and marine life cycle stages, although it is difficult to predict *a priori* at which stage the largest effects might be found.

## Materials and methods

### Study system and experimental groups

**Figure 1:**
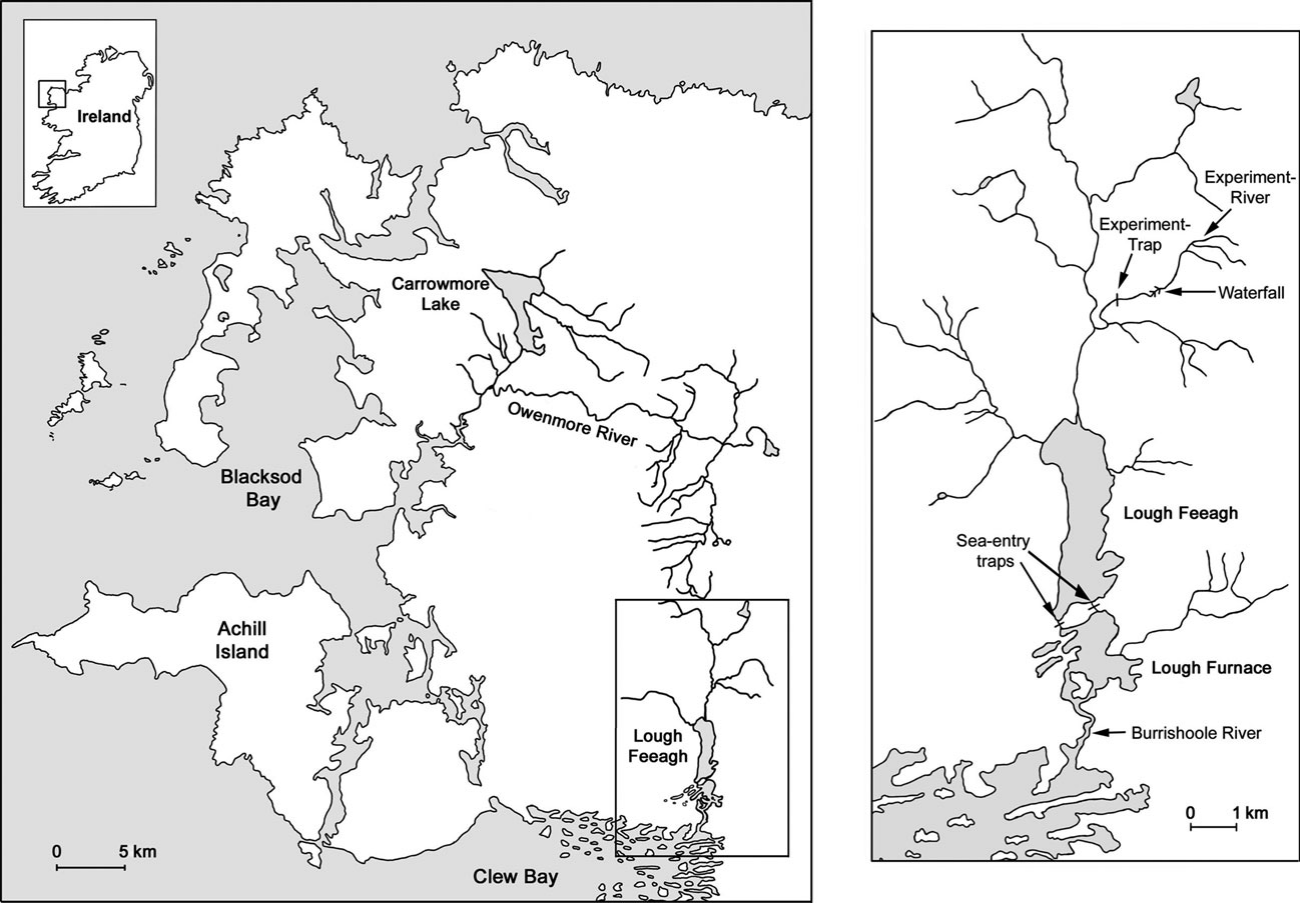
The Burrishoole (Local) and Owenmore (Foreign) catchments (left panel) and the location of the Srahrevagh River (experiment-river) trap and the sea-entry traps within the Burrishoole catchment, i.e. the ‘home’ environment (right panel).

The experiment was undertaken in the Burrishoole catchment (hereafter the ‘home environment’) in County Mayo in the west of Ireland (McGinnity et al. 1997, 2003, 2004; Byrne et al. 2003). An afferent river in the catchment (the Srahrevagh River, hereafter simply ‘experiment-river’) was utilized for the freshwater phase of the experiment and was equipped with a trap capable of capturing all downstream migrating juveniles and upstream migrating adults (experiment-trap). Two outlets from Lough Feeagh feed the tidal, semi-haline Lough Furnace, both of which have permanent upstream and downstream trapping facilities (sea-entry traps). The mouth of the neighbouring Owenmore River is approximately 50 km (in coastal distance) from the outflow of the home catchment, but both river systems have tributaries rising within 0.5 km of each other on the same mountain (Fig.1). Based on neutral microsatellite markers, De Eyto et al. (2011) reported significant genetic differences (*F*_ST_ = 0.0316, *P* < 0.05) between the Owenmore and Burrishoole *S. salar* populations.

Between 2007 and 2014, the run of Atlantic salmon in the Owenmore River ranged from 4074 in 2010 to 1308 in 2012 (Anon 2015 Report of the Standing Scientific Committee) comprising predominantly 1SW salmon (approx. 90% based on the component of the run after the 31st of May) and with a late-running, large grilse component. In 2008, the year in which broodstock were recovered, the estimated run size was 2460 salmon. The neighbouring Carrowmore system shares the same river mouth as the Owenmore system and has an average run of 1690 salmon with over 50% of the run occurring prior to the 31st of May. While both rivers have very distinct phenotypic differences, there is not believed to be a significant interaction between the two populations and the broodstock for this experiment did not include any Carrowmore fish. A small stocking programme was undertaken in the Owenmore River between 1974 and 2006 to counter the effects of sediment in parts of the rivers from peat harvesting, whereby 30–40 female fish and approximate equal number of males would be collected from the river in late November. These fish would then be stripped to produce between 100 000 and 150 000 eggs, which were then redistributed into nonimpacted parts of the river at the eyed-egg developmental stage. Using high and low survival from egg to smolt (1% and 0.33%, Burrishoole Annual Report 2011) and smolt to adult return rates (10% and 4%) derived from the Burrishoole salmon census programme, between 5 and 150 adult salmon would have been expected to return to the Owenmore in 2008 from the hatchery release in 2006, representing between 0.2 and 6.1% of the total estimated return to the river that year. On the basis of the small number of returning adult hatchery fish relative to the general population, the hatchery operation likely had a negligible impact on the genetic integrity of the Owenmore population. Until recently the Owenmore population was considered as one of Ireland's most pristine wild populations on the basis of minimum hatchery interference and superb freshwater habitat (excluding the river section affected by the peat silt). In the last 4 years a substantial decline (circa 50%) in numbers of adult fish returning to the river has become apparent (Anon 2015), attributed to poor marine survival, eliciting concern as to the river's conservation status. Similarly severe declines in the numbers of Burrishoole fish returning to the Burrishoole system have also been observed in recent years.

The annual number of adult wild salmon spawning in the Burrrishoole system has ranged from 203 to 1485 individuals, with <10% of these comprising multi-sea winter fish. The annual number of potential spawners of ranch origin has varied between 8 and 439. The Burrishoole population has had a long potential exposure to captive bred fish, which were derived from the local wild population. The history of the programme has been described in detail in McGinnity et al. (2009). In summary, a captive breeding programme for production of smolts for release and ranching was established from wild fish collected from the Burrishoole River between 1960 and 1964. Additional wild fish were included in the breeding stock between 1970 and 1975. The hatchery breeding population has been effectively closed since that time, with brood fish being selected from returning ranched fish. Since 1997, only a limited number of hatchery reared salmon are allowed enter the Burrishoole system (circa 100 fish). The vast majority of these return to the traps unspawned (over 80%) with only very few ranched kelts being recorded subsequently in the traps. Recent unpublished molecular data suggest that the Burrishoole ranch and Burrishoole wild populations are, at least at the molecular level, very different and that there has been very little change in the genetic composition of the Burrishoole wild population over time (P. McGinnity, pers. comm).

Hereafter, the Burrishoole population is referred to as ‘Local’ and the Owenmore population as ‘Foreign’.

Mature adult salmon were collected from the Foreign population by electrofishing during November 2008, and held at the Inland Fisheries Ireland brood stock holding facility at Glencullin, Bangor Erris until gamete stripping. Returning mature adults to the experiment-river within the home environment of the Local population were collected at the experiment-trap during December 2008 and held at the Marine Institute hatchery facility where experimental families were produced. While an attempt was made to collect only 1SW (one sea winter, i.e. grilse) fish in both cases (as they form the majority of the returning adult populations in both catchments and this follows the methods of McGinnity et al. (2004)), some 2SW fish were used as broodstock due to insufficient availability of 1SW fish (scale samples were used to confirm fish ages, full broodstock details are given in Appendix S1).

### Hatchery phase

Both Foreign and Local broodstock were stripped at the same time and Foreign milt and ova transported to the hatchery in containers. Experimental families were produced by artificial fertilization via a series of reciprocal crosses. Each Local female was crossed with one Local male and one Foreign male, and vice versa for each Foreign female, to produce a total of 52 full-sib families (nested within half-sib families), comprising four experimental groups (Table1; 1: Local_female_ × Local_male_, 2: Local_female_ × Foreign_male_, 3: Foreign_female_ × Local_male_, 4: Foreign_female_ × Foreign_male_ each consisting of 13 families). Owing to variations in the rate at which broodstock became ripe, it was not possible to produce all experimental families on the same day. The majority of the fish were stripped and the eggs fertilized on December 22nd 2008 (34 out of 52 families), with a further 14 families being created on December 29th 2008 and the remaining four families created on January 14th 2009. Each of 26 dams was mated twice, whereas out of 25 sires, 21 were mated twice, two were mated once and two were mated four times (Appendix S1). Local and Foreign dams did not differ significantly in fork length *L*_*F*_, mass-specific fecundity or eyed-egg volume (Appendix S1). Foreign 1SW sires were larger than Local 1SW sires (Appendix S1). Genetic samples (gill) were taken from each broodstock adult and retained for downstream parentage analyses.

**Table 1:** Groups of Atlantic salmon used in the experiment

Group	Number of dams*	Number of sires*	Number of families	Eyed-eggs to river	Eggs retained in hatchery	Ranched smolts to sea†
Local_female_ × Local_male_	13	11	13	13 640	3266	2361
Local_female_ × Foreign_male_	13	13	13	13 312	3343	2416
Foreign_female_ × Local_male_	13	13	13‡	13 280	3219	2327
Foreign_female_ × Foreign_male_	13	13	13‡	13 254	2778	2008

* Dams and sires were mated twice, with two Local males being mated four times each, see Appendix S1.

† Estimated number based on initial egg numbers per group, assuming equal egg-smolt survival.

‡ These groups each contained one family that exhibited anomalously low egg to alevin survival in the hatchery and representation analyses were conducted both including and excluding their eyed eggs. The ‘eggs retained in the hatchery column’ excludes these two families.

Fertilized eggs were placed in separate numbered trays in tanks in the hatchery and incubated to the ‘eyed-egg’ developmental stage, with dead eggs being removed and recorded on a regular basis. Eggs were ‘shocked’ at the eyed stage, a method used to identify nonviable eggs. Eyed ova from each family were counted volumetrically and assigned randomly to either the river (i.e. planted out) or to the hatchery (i.e. to produce ranched smolts). Prior to distributing ova to the river or hatchery, the volume of 200 eyed eggs (mls per 200 ova) was measured for each family and the total number of eyed ova available per family determined volumetrically. Appendix S1 provides a breakdown of egg numbers per family retained in the hatchery and planted out to the experimental stretch. An estimate of mean eyed-egg diameter for each family was also obtained by measuring the length of 25 eggs aligned on a V-shaped rule. These ova were retained in the hatchery in sectioned vibert boxes (one family per section) and their development checked every 2 days until full yolk sac absorption was reached, to check for unusual rates of mortalities or deformities. Families were mixed and disinfected immediately prior to transfer to the river. The date of transfer to the river was determined with a view to ensuring eggs were in place 2–3 weeks prior to hatching.

### Freshwater life stage

Eyed ova from 52 families, with the number of eggs varying among families from 380 to 1333 (Appendix S1), were planted in the experiment-river in March 2009. At this time, a random sub-sample from each family was retained in the hatchery and on-grown for the smolt release element of the study (Appendix S1). Early planted families (2nd March 2009, all derived from the early crosses (i.e. 22 December 2008), mid planted families (9th March 2009) derived from crosses made on 29th December 2008 and late-planted families (16th and 20th March 2009) derived from the late crosses (i.e. 14 January 2009). Eggs from all families were first mixed together in the hatchery and then batches of approximately 1000 ova were counted out into plastic wallets. Between five and six plastic wallets were supported in a box frame and boxes were placed across 11 artificial redds (one box per redd, with a total number of eggs per redd varying between 5000 and 6000), constructed according to Donaghy and Verspoor (2000). The total number of eggs planted out was approximately 51 500. The artificial redds were placed along a 2 km stretch of the experiment-river, consisting of 7250 m^2^ of salmonid habitat, bordered at one end by a series of impassable waterfalls and at the other by the experiment-trap capable of capturing salmon of all ages. To prevent natural spawning in the experiment-river, screens were deployed in late November 2008, to prevent adult salmon from moving upstream through the experiment-trap. However, it is possible that a limited number of early spawners could have accessed the experimental stretch and spawned naturally.

Monitoring of downstream movements at the experiment-trap began on the 22nd April 2009 and continued daily from that date onwards until May 2012 (by which time fish from the experimental families were 3+ smolts). All salmon captured at the trap were euthanized, *L*_*F*_ and mass measured and a tissue sample preserved in 99% molecular grade ethanol for subsequent parentage assignments. On the night of the 2nd July 2009, a large rainstorm caused catastrophic flooding in the Srahrevagh River. The experiment-trap was inundated for a period of 12 h with the river being diverted into neighbouring fields overnight. Large amounts of debris, including uprooted trees, gravel and silt, were washed downstream and lodged against the trap screens. This material was removed with the help of heavy machinery within a few hours, rendering the trap fully functional immediately thereafter. No fish were captured in the trap for a period of 36 h following the flood, despite it being operational. A large number of 0+ fry were captured (*n* = 1278) in the subsequent 5 days, estimated to represent approximately 35% of the population present in the experiment-river prior to the flood (C. O'Toole, unpublished data). This large migration of fry, presumably caused by the flood, may then have limited our subsequent ability to detect survival differences among groups. The experiment-river was electrofished on the 9th and 10th July, 2009, using a three pass method (Zippin 1958) to estimate the population density of 0+ salmon remaining in that portion of the river upstream of the trap. All 0+ salmon collected (*n* = 145) during electrofishing were tissue sampled for parentage assignments.

Wild smolts produced in the experiment river were enumerated as they emigrated through the experiment-trap, but not through the sea-entry traps. Any intervening mortality was likely to be minimal, given the relatively short and simple migration involved, and we assumed this was equal across groups when calculating lifetime success (see below). Our goal was not to accurately estimate absolute survival rates at each stage, but rather to make reasonable inferences regarding relative survival rate (and lifetime success) differences.

### Marine life stage

Since insufficient adult returns would have been obtained from the numbers of smolts likely to be produced in the experiment-river, the marine phase of the life cycle was examined by producing smolts in the hatchery, releasing them to sea and recovering the adult returns in the upstream traps (i.e. ranching). A total of 12 606 eyed eggs were held in the hatchery for on-growing to 1+ year old smolts (S1 smolts). Batches of fry from three stripping dates (see Appendix S1) were reared separately in 2 m circular tanks for first feeding and were transferred into a single 3.6 m circular tank on 18th June 2009. Salmon were graded on 5th August 2009 when large, medium and small grades were separated into three 3.6 m tanks. Medium and small grades were re-graded during October and November to recover any remaining potential 1+ smolts. Small grade fish remaining in November (which were too small to become S1 smolts) were euthanized. In February 2010, salmon presmolts were adipose finclipped, microtagged and cold branded as part of the National Salmon Microtagging and Tag Recovery Programme (Browne 1982; Ó Maoiléidigh et al. 1994; Wilkins et al. 2001). Finclipping was used to distinguish wild and ranched salmon in the upstream traps and cold branding was used to identify the experimental group. Salmon smolts were sampled prior to release (length, weight, tag retention) and tag retention was found to be 100%. Smolts were released into tidal Lough Furnace with other microtagged ranch groups on 30th April 2010. As the experimental population was managed as a single group within the hatchery, it was not possible to know precisely the group and family composition of the ranched smolts on release, although stock survival was high (95%). However, prior to release, a small piece of tail fin was clipped from a sub-sample of 400 fish to provide material for genetic analysis, of which 381 were genotyped to enable parentage assignment and to determine the group composition of the experimental release. At the time of sampling in March 2010, an additional microtag group of surplus Owenmore presmolts (1002 fish) had been added to the experimental group. Based on the proportions of the four groups at the eyed egg stage, assuming no differences in survival and smolting rates between the groups and accounting for the additional Owenmore presmolts, we had an expectation of 88, 90, 87 and 116 fish (for groups Local_female_ × Local_male_, Local_female_ × Foreign_male_, Foreign_female_ × Local_male_, and Foreign_female_ × Foreign_male_, respectively) in the sample of 381. The genetic analysis indicated almost identical observed numbers – 88, 89, 83 and 121 – to the expected (G test: *P* > 0.5). On this basis it was assumed that egg to smolt survival rates per group were identical in all groups and the number of ranched smolts per experimental group was therefore estimated by multiplying the number of initial (hatchery-retained) eyed eggs per group (Table1) by the overall egg to S1 smolt survival rate (9115 S1 smolts ÷ 12 606 eggs = 0.723). Returning mature fish (*n* = 134, of which 130 were sampled) from the 2010 release (1.4%) were recaptured at the sea-entry traps during the summer and autumn of 2011 as 1SW fish. A smaller number (*n* = 19) of 2SW fish returned to the traps the following year in 2012. All ranched salmon were culled in the trap and processed in the laboratory. Length, weight and sex were recorded, fish were cored to recover the microtag and scale and genetic samples collected.

### Microsatellite DNA profiling

Genomic DNA was extracted from biopsy tissue for all fish (*n* = 884 parr, *n* = 110 smolts and *n* = 149 returning adults) retained in the different stages of the study. Resulting DNA from all individuals were used as template to screen for variation at eight microsatellite loci: *Sp2210, Sp2216, Sp3016* (Paterson et al. 2004), *Ssa197, Ssa171*, (O'Reilly et al. 1996), SSOSL85 (Slettan et al. 1995), *SSaD170*, (EMBL accession number: AF525205) and *SsaD71* (King et al. 2005). Details on the methodological laboratory protocols used for genomic DNA extraction, microsatellite PCR amplification and allele genotyping are given in Appendix S2.

### Parentage assignment

Parentage assignment to family and experimental groups (i.e. native, non-native and hybrid) was carried out with the Family Assignment Program (FAP; Taggart 2007). FAP is particularly useful to estimate exclusion-based family assignment probabilities within family mixtures where all parental genotypes are known. In addition to parentage assignment, FAP also implements a predictive function that allows for users to assess the power of a given set of markers to correctly assign individuals to family. This feature was used to test the resolving power of the microsatellite maker loci used in this study to correctly assign parr, smolts and adults to the experimental families/groups. For parentage assignment (i.e. assignment analysis mode within FAP), the ‘allele size tolerance’ was set to zero while the ‘allele mismatch tolerance’ parameter was set to two. This allowed for an empirical evaluation of potential genotype scoring errors. Thus, in cases where mismatches were observed for one or two of the full set of marker loci, and taking the likelihood of full matches for the remaining loci (from the results of the power analysis), it was possible to account and to correct for mismatches resulting from scoring errors.

### Statistical analysis

#### Representation

As the group-specific counts in some samples are determined by both migration and survival (i.e. some fish may have migrated, rather than died), following McGinnity et al. (1997, 2003, 2004), counts are referred to as ‘representation’. G-tests for goodness-of-fit using Williams’ correction factor (Sokal and Rohlf 1995) were used to test for representation differences, with each non-native or hybrid group being compared in separate tests to the native (i.e. Local_female_ × Local_male_) group (all G tests were two-tailed and had one degree of freedom). The expected numbers for each group *i* were calculated as: 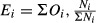, where *O*_*i*_ was the observed representation for group *i* and *N*_*i*_ was the number of eyed-eggs planted out for that group (when comparing representation at freshwater life stages), or the estimated number of ranched smolts for that group (when comparing representation of adult returns). G-tests assume that individual observations are independent (Sokal and Rohlf 1995), i.e. that the chances of an individual fish being represented in a given sample are independent of those of other individuals. This assumption may be violated slightly with these data, given that individuals sharing a mother or father may have correlated survival chances or migratory behaviours (due to the effects of shared genes and potential trans-generational environmental effects). To account for this potential nonindependence resulting from family structure and associated possible maternal effects, group-level differences in representation were also tested for using generalized linear mixed-effects models (GLMMs) with a negative binomial error distribution and a log link function, and random effects of dam and sire (nested within dam). The response variable was the number of represented individuals per full-sib family whilst the natural logarithm of the number of eyed-eggs planted per family was included as an offset variable (Zuur et al. 2009). Fixed effects of group, mean eyed-egg diameter, dam *L*_*F*_ and date of egg-planting were included. Results of the representation GLMMs (Appendix S3) were qualitatively consistent with those of the G-tests and for clarity only the latter are presented.

#### Offspring size

Variation in the length and mass of individual offspring was also examined in separate analyses for each of the five life/sampling stages. Linear mixed effects models (LMMs) were used assuming normally distributed errors and including dam and sire as random effects. Fixed effects of group: mean eyed-egg diameter, dam *L*_*F*_, date of egg-planting and, where appropriate, date of capture and its square (to capture nonlinear growth patterns) were included as candidate explanatory variables in all models. Date of egg-planting was a three level factor: ‘early’ = 2nd March 2009 (65% of families), ‘mid’ = 9th March 2009 (27% of families), ‘late’ = 16th and 20th March 2009 (8% of families). Since egg diameter and egg volume were highly correlated (*r* = 0.87) only egg diameter was included in the LMMs to avoid problems associated with multicollinearity (the correlation between dam *L*_*F*_ and mean eyed-egg diameter was 0.32). Nonsignificant fixed effects (as determined by dropping terms one at a time and using a likelihood ratio test (LRTs) to compare nested models fit by maximum likelihood) were removed in turn to identify the minimum adequate model (Zuur et al. 2009). Results were in all cases robust to different random effect structures (dropping dam or sire or both, structuring the residual variance by group or leaving unstructured). LMMs were fit in R version 3.0.2 (R Core Development Team 2008) using the lme function from the nlme package (Pinheiro et al. 2013), and all continuous covariates were first *z*-standardised.

#### Variance in family size

Gilk et al. (2004) hypothesized that hybridization between reproductively isolated populations may increase the variability in family size, if certain hybrid families contribute disproportionately to the total number of surviving offspring. They tested this by comparing the observed distribution of hybrid family sizes to that expected under a Poisson distribution with the same mean. If survival is entirely random, the index of variability in family size (*σ*^2^/*μ*, where *σ*^2^ is the variance in the number of offspring per family and *μ* is the mean number per family) tends to 1 (Crow and Morton 1955). We explored this possibility in a slightly different away. First, the adult returns data were split into a ‘pure’ group (Local_female_ × Local_male_ and Foreign_female_ × Foreign_male_) and a ‘hybrids’ group (Local_female_ × Foreign_male_ and Foreign_female_ × Local_male_). Next, both Poisson and negative binomial GLMs were fit to the data from each of these groups, including only an intercept in each case. Finally, an LRT was used to determine which error distribution better described the data. The Poisson (variance = mean = *μ*) is nested within the negative binomial distribution (variance = *μ *+ *αμ*^2^, where *α* = dispersion parameter) hence a LRT can be used to compare their relative fits (Zuur et al. 2009). If the negative binomial better describes the data (*α* ≠ 0), this indicates that the residual variance is greater than the mean (*σ*^2^/*μ *> 1), consistent with nonrandom variation in survival.

#### Overall lifetime success

Estimates of the lifetime success of each group, defined as the average number of green-eggs produced (i.e. at the beginning of the next generation) per eyed-egg planted out, were calculated following a series of steps described in Table3 (see Appendix S4 for full details). The absolute lifetime success of each group was divided by the lifetime success of the Local_female_ × Local_male_ group to obtain estimates relative to ‘pure natives’. Suitable habitat for juvenile salmonids is present in the river downstream of the experiment-trap and in freshwater Lough Feeagh. Thus, parr emigrating from the experiment-river would potentially be able to survive and produce smolts. A second measure of relative lifetime success was calculated assuming that emigrant parr had the same survival downstream as parr of the equivalent group remaining in the experiment-river.

## Results

### Microsatellite DNA profiling

Microsatellite multilocus genotypes were successfully obtained for 1143 individuals. PCR amplification success, over multiple loci per individual, was high, 1096 (96%) of specimens amplifying for all eight of the screened loci. Within microsatellite loci, genotyping reliability and consistency was also high. Thus, in each case alleles were clearly typed with minimum ambiguity. Double scoring by either by an independent operator or the same person on two different occasions (for 20% of genotypic data) confirmed data quality and consistency. Close comparison of control samples showed no problems calibrating genotypic data from the two screening platforms (i.e. ABI 3730XL and LI-COR DNA analysers).

### Parentage assignment

Results of power analysis (i.e. FAP predictive mode) indicates that the probability of correct assignment of a given individual to each of the 52 families and associated experimental groups, based on the full complement of microsatellite markers (*n* = 10) is 100%. That is, a full match unambiguously represents a true biological assignment to a particular family/experimental group. Since a number of nonamplifications was recorded among samples (i.e. nonamplification of one, two, three or four marker loci within the multi-locus genotype for a given individual), power analyses were independently carried out taking into consideration different combinations and/or number of markers to assess potential impacts on assignment reliability. Even for the few samples where genotypic data were only available for six of the 10 genotyped loci (i.e. 1% of all genotyped individuals), the probability of correct assignment to family was found to be invariably larger than 99.5% (depending on the particular marker combination involved) while the probability of assignment to group was always 100%. Of the 1143 putative offspring analysed, 878 (77%) were unambiguously assigned to single families. The remaining 265 fish (23%) did not assign to any of the experimental families. Given the number of allele mismatches observed over multiple loci, these nonassigned fish most likely represent offspring of a few early spawners that spawned naturally before the screens were deployed. Further details of fish assigned to family and/or experimental group is given in Table2 and the relevant sections below.

**Table 2:** Summary of number of individuals assigned or not back to family and experimental group for each life/sampling stage. Number of unique full-sib families given in parentheses. 1SW = one sea-winter adults. 2SW = two sea-winter adults

					1SW adult returns		2SW adult returns	
Group	Preflood emigrants	Flood emigrants	Postflood electro-fishing	Wild smolts	Female	Male	Female	Male
Local_female_ × Local_male_	28 (8)	72 (13)	22 (8)	14 (9)	23 (9)	34 (9)	6 (4)	
Local_female_ × Foreign_male_	45 (10)	94 (13)	33 (11)	11 (7)	7 (5)	12 (5)	4 (2)	2 (1)
Foreign_female_ × Local_male_	44 (9)	101 (12)	29 (10)	9 (7)	17 (5)	23 (6)	3 (3)	1 (1)
Foreign_female_ × Foreign_male_	83 (12)	109 (12)	22 (9)	16 (11)	7 (3)	7 (3)		
Total assigned	200 (39)	376 (50)	106 (38)	50 (34)	54 (22)	76 (23)	13 (9)	3 (2)
Not assigned	97	66	39	60	0	0	3	
Total sampled	297	442	145	110	54	76	19	

### Hatchery phase

Fertilization to eyed-egg survival in the hatchery sample was poorest for eggs produced by Foreign females, whether crossed with a Foreign male (77.7%, SE = ±3.03) or Local male (81.5 ± 3.18%, most likely because Foreign ova were transported to the hatchery and therefore experienced extra handling. This compared with 87.2% ± 3.80% survival at this stage for the pure Local cross and 87.9 ± 3.26% for the Local_female_ × Foreign_male_ cross. Results from a binomial GLMM, including dam and sire as random effects, indicated that these group differences in Foreign_female_ × Foreign_male_ fertilization to eyed-egg survival were not statistically significant from that of Local_female_ × Local_male_ (see Appendix S5 for details). No significant differences in fertilization to eyed-egg survival were found with respect to stripping date, dam life-history, dam fork-length, sire life-history and eyed-egg diameter (Appendix S5). No families exhibited total mortality at this stage.

Of the 25 subsampled eyed-eggs per family retained in the hatchery, survival to the alevin stage was very high overall (on average 24.28 alevins surviving per family), save for one Foreign_female_ × Local_male_ family (Family 39) where only 14 alevins survived, and another Foreign_female_ × Foreign_male_ family (Family 52) where only one alevin survived. Both families had been established during the third stripping event on January 14th 2009 and shared the same Foreign mother (OF_13). Survival rates in the hatchery from fertilization to eyed-egg were relatively high (slightly above average) for these two families, implying reduced viability at the alevin stage. None of the offspring sampled in the wild assigned back to either family, whereas all other families were represented at some life stage. The representation analyses were therefore repeated excluding these two families to test whether the results were affected, given that alevin viability might have been similarly low for these families in the experiment-stream (i.e. not reflective of LA *per se*). When calculating the expected number of ranched smolts per experimental group, the eggs from these two families were excluded. All other families showed no unusual rates of mortality or deformity in the hatchery subsamples.

### Freshwater life stage

#### Preflood emigrants

**Figure 2:**
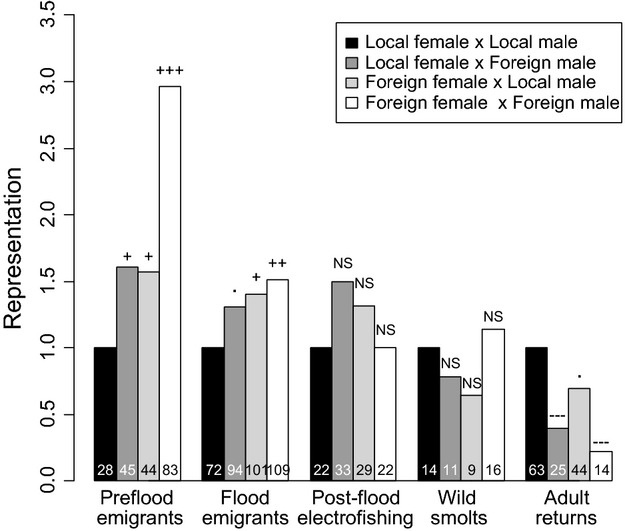
Representation of each group at each life/sampling stage. Representation values are relative to the Local_female_ × Local_male_ group in each case. Absolute representations (counts per group) are given in the bars. Significance of comparisons (based on G-tests) with Local_female_ × Local_male_ group indicated above bars: NS, nonsignificant; -, significantly less, +, significantly more; ·, 0.1 > *P > *0.05; -/+, 0.05 > *P* > 0.01; –/+=, 0.01 > *P* > 0.001, —/+++, *P* < 0.001.

During the period from the start of experiment-trap operation on the 22nd April 2009 until the 2nd July 2009 (i.e. the preflood trap sample), 412 0+ parr migrated in to the downstream trap. Of these, a random subset of 297 was genotyped and of those, 200 (67%) were successfully assigned parentage. The remaining 97 (33%) unassigned offspring were inferred to be Local nonexperimental (see Parentage Assignment section above). Almost three times as many (83 vs 28) Foreign_female_ × Foreign_male_ parr were caught in the trap prior to the flood compared with Local_female_ × Local_male_ parr (Fig.2; *G* = 30.0, *P* < 0.001). Of the hybrids, representation was higher in both the Local_female_ × Foreign_male_ group (*n* = 45 0+ parr; *G* = 4.4, *P* = 0.036) and the Foreign_female_ × Local_male_ group (*n* = 44 0+ parr; *G* = 4.0, *P* = 0.046) compared with the Local_female_ × Local_male_ group (Fig.2). No 0+ parr originating from late-planted families were represented in the preflood trap sample. Neither mean eyed-egg diameter nor dam *L*_*F*_ had a significant effect on family-level representation in this sample, or in any of the other life/sampling stages (Appendix S3). No significant differences (*P *>* *0.05) in parr *L*_*F*_ or parr mass were found with respect to group, nor was there any effect of dam *L*_*F*_ on parr *L*_*F*_ or parr mass. Mean eyed-egg diameter had a positive effect on parr *L*_*F*_ (Fig. S1; LMM: slope = 0.05 ± 0.01, *P *=* *0.001), as did date of capture (slope = 0.24 ± 0.016, *P *=* *0.001) and its square (0.09 ± 0.02, *P *=* *0.001). Parr originating from mid-planted families were smaller (3.09 ± 0.15 cm) than those from early-planted families (3.18 ± 0.15 cm; overall effect of date of egg-planting in LMM: *P *<* *0.001). For parr mass, only date of capture and its square had significant effects (both *P *<* *0.001). There was no significant difference in parr *L*_*F*_ between unassigned (wild-spawned, nonexperimental) and assigned (experimental) offspring in the preflood trap sample (*F*_1,295_ = 0.85; *P *=* *0.358), although the assigned offspring were slightly heavier (0.31 ± 0.01 g) than the unassigned (0.28 ± 0.01 g; *F*_1,295_* *= 5.05; *P* = 0.025).

#### Flood emigrants

During the 5 days following the flood, a total of 1278 0+ parr were captured migrating down through the experiment-trap. Of these, a random subset of 442 (34.5%) was genotyped, and of those, 376 (85%) were successfully assigned parentage. The remaining 66 unassigned offspring were also likely to have been produced by naturally spawning, nonexperimental, Local native parents. Approximately 1.5 times as many (109 vs 72) Foreign_female_ × Foreign_male_ parr were represented relative to Local_female_ × Local_male_ parr (Fig.2; *G* = 8.9, *P* = 0.003). Representation was higher in both hybrid groups (Local_female_ × Foreign_male_: 94 0+ parr; *G* = 3.5, *P* = 0.062; Foreign_female_ × Local_male_ group: 101 0+ parr; *G* = 5.7, *P *=* *0.017) compared with the Local_female_ × Local_male_ group (Fig.2). Fewer parr were represented from mid-planted families (mean 6.5 per family) and late-planted families (mean 1.0) relative to early-planted families (mean 8.3 per family).

Among the flood migrants, significant (*P *=* *0.042) differences in parr *L*_*F*_ were found with respect to group (Local_female_ × Local_male_ = 4.01 ± 0.06 cm; Local_female_ × Foreign_male_ = 4.10 ± 0.04 cm; Foreign_female_ × Local_male_ = 4.00 ± 0.04 cm; Foreign_female_ × Foreign_male_ = 4.08 ± 0.04 cm). Dam *L*_*F*_ had a significant positive effect (Fig. S1; slope = 0.10 ± 0.03, *P *=* *0.001) on parr *L*_*F*_ in this sample but there was no effect of mean eyed-egg diameter. Date of egg-planting had a significant overall effect on parr *L*_*F*_ (*P *<* *0.001), with parr from late-planted families being smaller (3.42 ± 0.01 cm) than those from mid- (3.94 ± 0.04 cm) or early-planted (4.01 ± 0.03 cm) families. Groups differed significantly in parr mass (*P = *0.008; mean mass for Local_female_ × Local_male_ = 0.67 ± 0.03 g; Local_female_ × Foreign_male_ = 0.71 ± 0.03 g; Foreign_female_ × Local_male_ = 0.70 ± 0.02 g; Foreign_female_ × Foreign_male_ = 0.75 ± 0.02 g). Mean eyed-egg diameter (Fig. S1; slope = 0.05 ± 0.02, *P *=* *0.012), dam *L*_*F*_ (Fig. S1; slope = 0.05 ± 0.02, *P *=* *0.009) and date of egg-planting (*P *=* *0.016; early families: 0.73 ± 0.01 g; mid families: 0.66 ± 0.02 g; late families: 0.43 ± 0.04 g) all had a significant effect on parr mass. There was no significant difference in parr *L*_*F*_ between unassigned (nonexperimental) and assigned (experimental) offspring in the postflood trap sample (*F*_1,440_* *= 0.90; *P *=* *0.344), or in parr mass (*F*_1,440_* *= 0.82; *P = *0.366).

#### Postflood electrofished parr

A sample of 176 0+ parr was obtained by electrofishing on 12th July 2009, 10 days after the flood. Of these, a random subset of 145 (82%) was genotyped, and of those, 106 (73%) were successfully assigned parentage. The remaining 39 unassigned offspring, as discussed above, were likely to have been produced by naturally spawning, nonexperimental, Local parents. An equal number (22) of Foreign_female_ × Foreign_male_ and Local_female_ × Local_male_ parr were represented (Fig.2). Representation was slightly higher in both hybrid groups (33 and 20 for Local_female_ × Foreign_male_ and Foreign_female_ × Local_male_, respectively) but neither was significantly over-represented relative to the Local_female_ × Local_male_ group (Local_female_ × Foreign_male_: *G *=* *2.5, *P *=* *0.116; Foreign_female_ × Local_male_: *G *=* *1.1, *P *=* *0.284). Fewer parr were represented from late-planted families (mean 0.25 per family) compared with early-planted (mean 1.93) and mid-planted families (mean 2.29 per family).

Among the electrofished parr, no significant differences in parr *L*_*F*_ or parr mass were found with respect to group. Dam *L*_*F*_ and date of egg-planting did not influence parr *L*_*F*_ or parr mass in this sample but mean eyed-egg diameter did have a significant positive effect on each (Fig. S1; parr *L*_*F*_: slope = 0.19 ± 0.04, *P *<* *0.001; parr mass: slope = 0.017 ± 0.003, *P *<* *0.001). There was no significant difference in parr *L*_*F*_ between unassigned (wild-spawned) and assigned (experimental) offspring in the electrofished sample (*F*_1,143_* *= 0.60; *P *=* *0.434), or in parr mass (*F*_1,143_* *= 2.29; *P = *0.133).

#### Wild smolts

Remaining migration from the experiment-river occurred in two phases: an ‘autumn’ migration of presmolts (*n* = 45) in the period from 2 November 2010 to 14 January 2011, and a typical ‘spring’ 2+ year smolt migration (*n* = 56) from 10 February to 5 May 2011. A further nine 3+ year smolts were captured in the experiment-trap in the spring of 2012. Of the autumn presmolts, 21 were successfully assigned parentage, with the remaining 24 likely to have been produced by wild (nonexperimental) spawners. Of the spring smolts, 29 were successfully assigned parentage, the remaining 36 likely to have been produced by wild spawners. Taking the autumn presmolts and spring 2+ and 3+ smolts together, the representation of groups (Fig.2) did not differ significantly from the Local_female_ × Local_male_ group (Local_female_ × Foreign_male_: *G *=* *0.3, *P *=* *0.593; Foreign_female_ × Local_male_: *G *=* *0.9, *P *=* *0.331; Foreign_female_ × Foreign_male_: *G *=* *0.2, *P *=* *0.660). No smolts were represented from late-planted families, compared with an average of 0.94 smolts per family from early- and 1.29 smolts per family from mid-planted families. The overall absolute survival of the experimental stream population from eyed-egg to smolt was estimated at 0.09%. Absolute survival of 0+ parr in September 2009 to the 1+ parr stage in August 2010 was estimated at 56.5%. The survival rate over the second winter from August 2010 to the smolt migration in spring 2011 was estimated at 7.0%.

No significant differences in smolt *L*_*F*_ or smolt mass were found with respect to group. Mean eyed-egg diameter, dam *L*_*F*_ and date of egg-planting did not influence smolt *L*_*F*_ or smolt mass. Spring smolts (mean *L*_*F*_ = 12.40 ± 0.16 cm) were larger than autumn presmolts (mean *L*_*F*_ = 11.62 ± 0.18 cm; *P *<* *0.001). There was no significant difference in smolt *L*_*F*_ between unassigned (wild-spawned) and assigned (experimental) offspring (*F*_1,108_* *= 2.74; *P *= 0.101), or in smolt mass (*F*_1,108_* *= 2.27; *P = *0.135).

### Marine life stage

**Figure 3:**
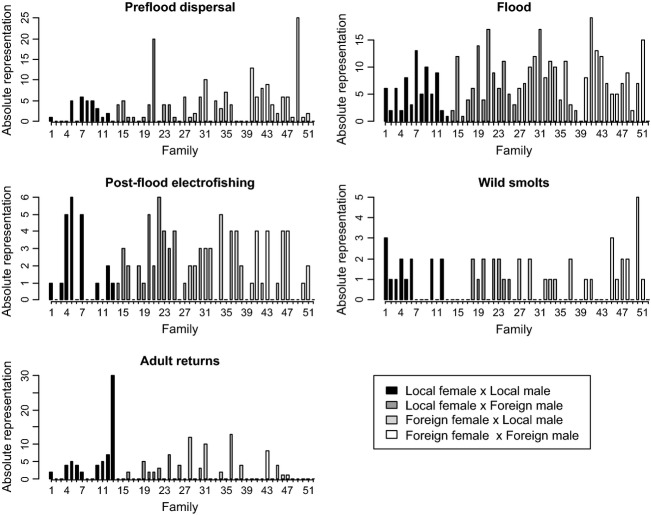
Absolute representation of each experimental group at each life/sampling stage, broken down by family. Note that unrepresented families (i.e. count = 0) are included in each panel.

The overall egg to hatchery 1+ smolt survival rate was estimated at 72.3% (9115 S1 smolts released/12 606 initial eyed eggs). Adult salmon (*n* = 149, with 146 being successfully assigned parentage) returned from the ocean after one and two winters at sea (1SW and 2SW) with 87.5% being 1SW. The Foreign_female_ × Foreign_male_ group was significantly under-represented (14 adults) relative to the Local_female_ × Local_male_ group (63 adults; Fig.2; *G = *26.1, *P < *0.001). There was also a deficit of Local_female_ × Foreign_male_ adults (25 fish) relative to pure natives (*G = *17.7, *P < *0.001). The second hybrid group, Foreign_female_ × Local_male_, had a representation rate of 69.8% (44 fish) relative to pure natives, although this difference was not statistically significant (*G = *3.1, *P *=* *0.078). Of the 16 fish that returned as 2SW adults and could be assigned back to families/groups, six belonged to the Local_female_ × Local_male_ group and four to the Foreign_female_ × Local_male_ group (the G test results were qualitatively unchanged when only 1SW adult returns were included). The overall sex ratio for 1SW was 1:1.4 (female:male) and 4.3:1 for 2 SW fish. Approximately equal numbers of male and female 1SW adults were represented in each group (G-test of independence to test for unequal sex ratios: *G *=* *0.13, *P *=* *0.97). Closer examination revealed one apparent ‘super family’ in the Local_female_ × Local_male_ group (Family 13), which produced 30 returning adults (almost half the total for this group, Fig.3). However, the number of eyed ova for this group was also high and thus the ratio of returns to initial eggs is not unusual (i.e. this family is not an outlier in terms of scaled representation) and G-test results were qualitatively the same when this family was omitted.

No significant differences in adult *L*_*F*_ or mass were found with respect to group. Dam *L*_*F*_ did not influence adult *L*_*F*_ or mass but there was a significant negative effect of mean eyed-egg diameter in both cases (Fig. S1; adult *L*_*F*_: slope = −0.83 ± 0.35; *P *=* *0.017; adult mass: slope = −107.5 ± 46.7, *P = *0.021; in both cases controlling for sea age and sex effects). Date of return to the catchment (i.e. date of capture in sea-entry traps) varied with respect to group, with Local_female_ × Local_male_ adults returning the earliest, Foreign_female_ × Foreign_male_ the latest and hybrid intermediate (overall group differences were marginally nonsignificant; LRT = 7.42, df = 3, *P *=* *0.06). 2SW fish also returned earlier than 1SW fish (LRT = 20.9, df = 1, *P *<* *0.001).

### Variance in family size

A negative binomial GLM better fit the adult returns data for pure families than a Poisson GLM (LRT = 115.5, df = 2, *P *<* *0.001). This result was robust to excluding Family 13. Similarly, a negative binomial GLM better fit the hybrids adult returns data than did a Poisson GLM (LRT = 65.0, df = 2, *P *<* *0.001). These findings are consistent with nonrandom marine survival of both pure (*α *= 0.30; *σ*^2^/*μ *= 10.7) and hybrid families (*α *= 0.37; *σ*^2^/*μ *= 8.2).

### Overall lifetime success

Lifetime success of Foreign_female_ × Foreign_male_ fish was estimated to be 31% that of native fish (Table3), while that of the hybrids was 36% and 44%, for Local_female_ × Foreign_male_ and Foreign_female_ × Local_male_, respectively. Under the assumption that parr migrants survived downstream of the experiment-trap, the relative lifetime success of the Foreign_female_ × Foreign_male_ group was estimated at 38% of the native group, while the relative lifetime successes of the Local_female_ × Foreign_male_ and Foreign_female_ × Local_male_ hybrid groups were estimated at 36% and 46%, respectively (Table3). These results were qualitatively unchanged when progeny from the two families that exhibited anomalously high alevin mortality in the hatchery (which derived from the same Foreign female) were excluded.

**Table 3:** Estimating lifetime success (eyed-egg to green-egg) of each group

Row	Known or estimated quantity	All groups	Local_female_ × Local_male_	Local_female_ × Foreign_male_	Foreign_female_ × Local_male_	Foreign_female_ × Foreign_male_
A	Number of returning adult females, *N*_*j*_	67	29	11	20	7
B	Mean mass (kg) of returning adult females	2.42	2.40	2.88	2.29	2.20
C	Total mass of returning adult females, kg (=A × B)	162.14	69.60	31.68	45.80	15.40
D	Mass specific fecundity, 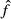 (eggs/kg)	1500	1500	1500	1500	1500
E	Total number of green-eggs produced by returning adult females (=C × D)	243 210	104 400	47 520	68 700	23 100
F	Estimated number of female ranched smolts*	4558	1181	1209	1164	1004
G	Estimated number of green-eggs per female smolt, given it survives to returning adult (=E/F)	53.36	88.42	39.32	59.03	23.00
H	Number of wild female smolts at experiment-trap†	25	7	5.5	4.5	8
I	Estimated number of wild female smolts at sea-entry traps, assuming parr emigrants survived‡	71.5	18.5	14.5	12.5	26.0
J	Estimated number of green-eggs for all wild smolts, assuming parr emigrants do not survive (=G × H)	1334	619	216	266	184
K	Estimated number of green-eggs for all wild smolts, assuming parr emigrants survive (=G × I)	3816	1636	570	738	598
L	Initial number of eyed-eggs planted out	53 486	13 640	13 312	13 280	13 254
M	Absolute lifetime success, assuming parr emigrants do not survive (J/L)	0.0249	0.0454	0.0162	0.0200	0.0139
N	Absolute lifetime success, assuming parr emigrants survive (K/L)	0.0713	0.1199	0.0428	0.0556	0.0451
O	Relative lifetime success, assuming parr emigrants do not survive		1.00	0.36	0.44	0.31
P	Relative lifetime success, assuming parr emigrants survive		1.00	0.36	0.46	0.38

* Estimated based on initial egg numbers, assuming equal egg-smolt survival and equal sex ratio.

† Actual number of smolts × 0.5, assuming an equal sex ratio.

‡ Parr emigrants assumed to survive at same rate as parr belonging to same group that did not migrate from the experiment-river.

## Discussion

The overall lifetime success of Foreign fish was estimated to be only 31% that of Local fish under communal rearing conditions (38% if emigrating parr were assumed to survive), which is similar to the value of 35% reported in a previous experiment by McGinnity et al. (2004). These results are consistent with LA of a large magnitude occurring between geographically adjacent rivers, despite potential for gene flow between them, and that this LA is temporally stable (at least between experiments conducted a decade apart). However, while we have demonstrated higher fitness of ‘local’ over ‘foreign’ in the home environment of the local strain, we did not perform full reciprocal transplants and hence could not assess the home versus away criterion for LA (Kawecki and Ebert 2004). We therefore cannot rule out the possibility that Local fish would have also outperformed Foreign fish in the ‘away’ (i.e. Owenmore River) environment (i.e. they are a superior strain in all environments), although we see no obvious reason why that should be the case. In addition to confirming the marine performance differences also found by McGinnity et al. (2004), the novel aspect of the current study was that reciprocal hybrids between Local and Foreign groups were included. This allowed for a more robust test of an additive genetic basis to among-population fitness differences (Gilk et al. 2004; Fraser et al. 2008; Aykanat et al. 2012). The average lifetime success of these hybrids (taking Local_female_ × Foreign_male_ and Foreign_female_ × Local_male_ together) was estimated to be some 40% that of the pure native group (41% if emigrating parr were assumed to survive). These values are approximate, given that a series of assumptions were made (Table3 and Appendix S4) and each intermediate calculation step was associated with (largely unquantifiable) error. We are nevertheless confident that they provide a reasonable snapshot of inherent relative fitness differences, since lifetime success differences were driven primarily by variation among groups in the numbers of returning adults and the latter was measured almost without error. The sea-entry traps capture all adults migrating back into the system and the genotypes of all original broodstock were known; hence parentage of returning adults was assigned with close to 100% accuracy, save for a small amount of possible genotyping error.

In general, the intermediate performance of hybrids relative to inferior Foreigns and superior Locals is strongly indicative of either LA or intrinsic outbreeding depression (Hatfield and Schluter 1999; Kawecki and Ebert 2004). Here, environmental contributions to fitness differences were eliminated as far as possible by the common garden design. Maternal or paternal effects may also contribute to population divergence in phenotypic traits involved in LA (Garcia de Leaniz et al. 2007; Fraser et al. 2011). For example, using a factorial half-sib breeding design coupled with common-garden rearing, Aykanat et al. (2012) found that maternal effects contributed more to phenotypic differences in size-at-age and early survival traits among Chinook salmon (*Onchorynchus tshawytscha*) populations in British Columbia than did additive genetic effects. However, their experiments were conducted in hatchery environments and as such the inferences may not hold in the wild (Einum and Fleming 1999). Aykanat et al. (2012) also did not examine marine survival variation, which is where we find the biggest performance differences. Maternal influences on offspring performance are known to be more important early in salmonid life histories (Heath et al. 1999; Garant et al. 2003) and hence are unlikely to account for the reduced marine performance we observed in the Foreign and hybrid groups. Moreover, dam *L*_*F*_ and mean-eyed egg diameter did not explain any of the among-family variation in representation at any life stage (Appendix S3). Egg size was found to positively affect the length and mass of electro-fished parr and emigrant parr, indicative of maternal effects on size-at-age, but unexpectedly we also found a weak negative relationship between (family-mean) egg size and the size of returning adults, controlling for variation due to sex and sea age (Fig. S1). This relationship could not be explained by matriline effects mediated via egg size, as there were no egg size differences between Local and Foreign dams. Finally, paternal effects have also been documented in salmonids (Heath et al. 1999) and more generally in fishes (Green 2008). In general, we did not find any evidence for paternal effects mediated via sire life-history (Appendix S3) on offspring survival, but an effect of patriline was evident for the adult returns data (see below).

Unlike McGinnity et al. (2004), who found reduced smolt output for the Foreign group relative to the Local group in the river, no group differences in smolt output were found in this study. This may reflect the fact that selective pressures experienced during the freshwater stage are variable across years, implying population × environment (likely due to genotype × environment) interactions in freshwater survival and highlighting the importance of repeating common garden experiments under a range of natural conditions. LA is more likely to result when selective pressures are temporally stable (Kawecki and Ebert 2004). The catastrophic flood event that occurred in 2009 may have been atypical in this regard and may have limited our ability to detect LA at parr stages, given that absolute egg-to-smolt survival rates (and hence wild smolt sample sizes) were extremely low for this experimental cohort (an order of magnitude lower than in previous experiments in the same system, McGinnity et al. 2004; De Eyto et al. 2011). Conceivably, the environment prior to the flood may be more representative of the selection pressures driving LA for the native population. At the same time, extreme flood events may have important long-term selective and genetic consequences (e.g. Pujolar et al. 2011) given the potentially long-lasting alternations to the physical and biotic structure of the stream rearing environment. Indeed, annual invertebrate surveys showed a change in invertebrate fauna in terms of both composition and abundance in the Srahrevagh River between 2010 and preceding years (E. de Eyto, pers. comm.). Fry dispersal behaviour was found to differ among the groups, with many more Foreign_female_ × Foreign_male_ fry captured moving downstream through the experiment-trap both prior to, and several days after, the flood event (Fig.2). McGinnity et al. (2004) similarly found that Owenmore (Foreign) parr were much more likely to move downstream than Local fish and speculated that downstream emigration may be adaptive in the Owenmore River, where most of the best rearing habitat is currently downstream of the spawning habitat. Potential rearing habitat is available downstream of the experiment-trap in the home environment of the Local group, either in the stream or in Lough Feeagh, but this may be sub-optimal relative to upstream rearing habitat and hence downstream dispersal may entail stronger fitness costs for Local juveniles. The almost exactly intermediate levels of dispersal found for both hybrids relative to the parental groups (Fig.2) are strongly consistent with an additive genetic basis to population differences in this behavioural trait; parr migration having previously been shown to be under genetic control in salmonids (Raleigh 1971). It was not possible, unfortunately, to monitor the subsequent survival of parr emigrants in this study to test the adaptive basis of these behavioural differences. Parr remaining in the stream (electro-fished sample) were significantly longer and heavier than flood emigrants (which were sampled only 5–10 days previously, and therefore not expected to differ that much in size simply due to age differences), implying that size is a significant factor affecting competitive displacement responses to extreme events. It is possible that larger fish were better able to maintain their territories within the stream both during and directly after the flood (Jonsson and Jonsson 2009), or to gain new territories following disturbances to physical habitat. The lack of size differences between Local and Foreign parr in the flood emigrant and postflood electro-fishing samples, however, suggests that dispersal behaviour differences *between groups* were not driven by size effects.

Marine survival of ranched smolts was substantially higher in Locals, with only 22% as many returning adults among the Foreign group and 55% as many hybrids (taking the average counts of Local_female_ × Foreign_male_ and Foreign_female_ × Foreign_male_). The additive expectation for the hybrids is 61% based on the mid-parent value, thus the slightly lower observed marine survival of 55% (mean of both hybrid groups together) may indicate some intrinsic outbreeding depression with an additive-dominance component (Lynch and Walsh 1998). Treating hybrid groups separately, however, the number of returning adults in the Foreign_female_ × Local_male_ group was not significantly different from the Local_female_ × Local_male_ group, whilst the numbers of Local_female_ × Foreign_male_ adults were not significantly different from the Foreign_female_ × Foreign_male_ group. This would seem to suggest that paternal line (i.e. population of origin of sires) had a strong effect on marine returns, whereas maternal line had no effect, which is difficult to reconcile with a purely additive genetic basis to population divergence. One possibility is that marine survival was actually similar for the two hybrid groups but homing ability differed and had an additive-dominance basis, with the dominance component driven by a paternal effect. For example, in an experiment where males from a native population of pink salmon (*Onchornynchus gorbuscha*) were crossed with females from a non-native population and the unfed fry were released in a stream in the native population's catchment, the hybrids exhibited similar marine survival as pure non-natives but returned in greater numbers to the natal stream, indicating a strong effect of patriline on homing tendencies (Bams 1976). Reciprocal hybrids (i.e. local female, foreign male) were not included in the study of Bams (1976), however, so it was not clear whether patrilineal effects on homing would have exceeded matrilineal effects, while subsequent hybridization experiments involving geographically distant pink salmon populations found similar homing rates in hybrids as in controls (Gilk et al. 2004). Other plausible explanations for the contrasting adult returns patterns observed in hybrids include sex-linkage and genetic imprinting. Sex-specific patterns of heterosis have been documented in laboratory mice (Hannon et al. 2011), but here we found no sex ratio differences between Local_female_ × Foreign_male_ and Foreign_female_ × Local_male_ hybrid groups in terms of adult returns, which would argue against sex-linked recessive mutations (or mutations with sex-specific expression) affecting marine performance.

The specific phenotypic traits driving the marine performance differences between the groups can also only be speculated at. Foreign smolts migrating naturally from their own home environment (i.e. the Owenmore River) have limited estuarine rearing or passage, as the Owenmore River more-or-less directly enters the sea. Local smolts, in contrast, must first pass through brackish Lough Furnace before entering full seawater. The pure Foreign and hybrid groups may therefore have lacked the appropriate adaptations for coping with the physical and/or biotic challenges posed by temporary residence in, and navigation out of, Lough Furnace (McGinnity et al. 2004). Local smolts must also migrate due west on leaving Lough Furnace to reach the open ocean, whereas Foreign smolts (in their own natal home environment) must first migrate in a south-westerly direction (McGinnity et al. 2004). Given that the bulk of marine mortality in Atlantic salmon is believed to occur during the first few weeks to months after smolting (Hansen et al. 2003), the initial transition to saltwater may be the period where selection is strongest. The large marine performance differences between Local and Foreign smolts released from the Burrishoole system are remarkable given that the mouths of each river system are only ∼50 km apart and fish from each population presumably experience very similar conditions once they move offshore. F1 inter-population hybrids of Atlantic salmon have previously been found to exhibit intermediate marine distributions compared with parental populations (Kallio-Nyberg et al. 2000) and it is thus conceivable that Foreign and Local fish migrate along different oceanic routes or to different feeding grounds (and therefore potentially experienced different mortality regimes). Alternatively, they may have similar migration pathways and differences in marine survival could have evolved as a byproduct (e.g. due to life history trade-offs) of evolutionary responses to divergent freshwater selection. We also found weak evidence for genetically based population divergence in return migration timing, with natives returning earlier than non-natives and hybrids intermediate.

Our findings add to a growing number of studies demonstrating marine performance differences between genetically divergent salmon populations (McGinnity et al. 1997, 2003, 2004; Gilk et al. 2004) and highlight the need to better understand the extent and scale of LA during the marine phase and potential linkages between freshwater and marine adaptations (Fraser et al. 2011). In addition to the present study, over the last 20 years or so, a number of common garden experiments have been conducted in the Burrishoole system comparing the relative performance of the progeny of native and non-native Atlantic salmon, including farm (Norwegian) v wild comparisons (McGinnity et al. 1997, 2003) and native Irish v non-native Irish (McGinnity et al. 2004) in both river and sea environments. While some biologically significant differences were observed among groups in each study in relation to performance in freshwater (juvenile survival, juvenile migratory behaviour, size at age, propensity for precocity in male parr), these were typically of limited magnitude. Survival differences among experimental groups varied between 20% and 40% and, in at least half of the studies, the progeny of the non-native fish (either pure or hybrid born) performed as well in the river and in some instances better than the local population. Significantly, however, in these same studies, it was in the marine environment where very large scale performance differences were found: adult return rates for the progeny of local wild fish were seven times that of farm fish (McGinnity et al. 2003); adult return rates for the progeny of local Burrishoole were nine times that of foreign Owenmore fish in the study by McGinnity et al. (2004); while in the current study, adult return rates were four times higher for Locals over Foreign fish.

This is an important insight regarding how we perceive the operation of LA in salmon and other diadromous fishes, particularly given that the opportunity for divergent natural selection is often assumed (e.g. Quinn 2005; Garcia de Leaniz et al. 2007) to be larger during freshwater life history phases than during marine life history phases. Based on studies of Atlantic salmon in Ireland (McGinnity et al. 2003, 2004; this study), it would appear that the traits associated with the marine environment or the transition between local river environments and marine environments (or indeed carry over effects from the freshwater environment that are important for life in the sea), are of substantially greater importance in respect of LA than the more obviously local factors in the river environment. Such traits may include ocean entry timing, predator avoidance and the ability to orientate into favourable ocean currents for transportation to feeding grounds. Likewise, a successful return to the natal river and arrival to the spawning grounds will be contingent on homing orientation, time spent at sea, timing of return and timing of river entry. In our case, the experimental groups may have differed genetically for traits affecting their ability to home back to the sea-entry traps beside the hatchery (which is where we then recaptured adult returns), although we had no way of quantifying this. All groups had ample opportunity to imprint on the local water source prior to release as smolts, as the hatchery is supplied with water directly from the outflow of Lough Feeagh. Moreover, homing to the native environment can itself be considered a LA (Quinn 2005). Thus, both differences in marine survival and differences in homing are consistent with genetically based LA at the marine phase, so long as one defines fitness locally (i.e. fitness = recruitment back into the natal population). If homing differed among groups but marine survival did not, then global fitness (i.e. recruitment to any population) may have then been similar among them. In our view, local fitness is a more relevant success metric in studies of LA given that adaptation to local conditions is expected to be reinforced by precise homing and diluted by straying and resultant gene flow. Finally, we also consider it unlikely that a longer history of captive breeding in the Local population rendered ranched smolts of Local parentage better adapted for ranching performance than smolts of Foreign parentage, for example related to superior homing abilities or ability to overcome in the ocean any deleterious developmental legacy induced in the hatchery. We only used wild fish for the experiment, so one would then have to assume that there has been sufficient gene flow from the hatchery population to the Local wild population to cause genetic changes to the latter, which in turn conferred superior capacity for marine survival or homing. Recent unpublished molecular data would suggest that the Local ranch and wild populations are, at least at the molecular level, very different and that there has been very little change in the genetic composition of the Local wild population over time (Philip McGinnity pers. comm).

### Conservation and management implications

The extent and scale of LA are crucial considerations from both conservation and wildlife management perspectives. For example, the success of translocation programs for threatened taxa may depend on the degree of adaptive matching of translocated individuals to their new environments (Allendorf and Waples 1996; Hufford and Mazer 2003; Weeks et al. 2011), while genetic rescue programs may do more harm than good if artificial immigrants are poorly adapted to local conditions in the recipient population (Tallmon et al. 2004; but see Whiteley et al. 2015). Forecasts of range shifts or species vulnerabilities in the face of climate change may also be altered substantially when adaptation to local climates is taken into account. This is because climate envelopes of differentiated populations are likely to be narrower than climate envelopes inferred at the species level (Aitken et al. 2008; Phillimore et al. 2010; Eliason et al. 2011). Supplemental stocking of native populations with non-native fish has been commonly practiced in Atlantic salmon and related species and may deliver demographic benefits in some situations. These benefits must be weighed against potential genetic risks associated with outbreeding depression and the latter might be assumed to be minimal when salmon from neighbouring rivers in the same region are used. Our results argue strongly against this, however, given that lifetime fitness was much lower for foreign salmon from a catchment only 50 km away (by coastal distance) relative to locals. Crucially, the reduced performance of hybrids (at least for one of the hybrid groups) relative to natives (see also Gilk et al. 2004) indicates that supplemental stocking could result in cumulative reductions in mean fitness in recipient populations if non-natives successfully interbreed with locally adapted natives. Thus, while LA may be an uncertain evolutionary outcome at intra-regional scales (e.g. <100–200 km) in salmonids due to the potentially homogenizing effects of inter-population straying (Adkison 1995; Fraser et al. 2011), a lack of LA at these scales should not be taken for granted, given that ‘microgeographic’ adaptation in the face of gene flow has been documented in salmonids (e.g. Westley et al. 2012) and other taxa (Richardson et al. 2014). While the consequences of outbreeding may be highly variable or uncertain when genetic distances between populations are small (Houde et al. 2011), our results show that large adaptive differences may exist between geographically proximate stocks despite modest neutral genetic differentiation. This highlights the danger of using measures such as *F*_ST_ to assess the evolutionary consequences of stocking programs. The precautionary principle would therefore suggest prudence and a full consideration of the risks of outbreeding depression before proceeding with stocking, even if broodstock are obtained from neighbouring catchments or tributaries within the same catchment.

## Data archiving statement

Data for this study are available at: http://dx.doi.org/10.5061/dryad.s171t.

## Supporting Information

**Figure S1.** Progeny fork length and mass versus mean eyed-egg diameter and dam fork length, plotted separately for each life/sampling stage.

**Appendix S1.** Broodstock details.

**Appendix S2.** Details of genomic DNA extraction, microsatellite PCR amplification and allele genotyping.

**Appendix S3.** Results of the representation GLMMs.

**Appendix S4.** Estimating lifetime success of each group

**Appendix S5.** Survival of subsampled eyed eggs retained in the hatchery from all families outplanted in the experimental river.

## References

[b1] Adkison MD (1995). Population differentiation in Pacific salmons: local adaptation genetic drift, or the environment?. Canadian Journal of Fisheries and Aquatic Sciences.

[b2] Aitken SN, Yeaman S, Holliday JA, Wang T, Curtis-McLane S (2008). Adaptation, migration or extirpation: climate change outcomes for tree populations. Evolutionary Applications.

[b3] Allendorf FW, Waples RS, Avise JC, Hamrick JL (1996). Conservation and genetics of salmonid fishes. Conservation Genetics, Case Histories from Nature.

[b4] Anon (2015).

[b5] Araki H, Berejikian BA, Ford MJ, Blouin MS (2008). Fitness of hatchery-reared salmonids in the wild. Evolutionary Applications.

[b6] Aykanat T, Bryden CA, Heath DD (2012). Sex-biased genetic component distribution among populations: additive genetic and maternal contributions to phenotypic differences among populations of Chinook salmon. Journal of Evolutionary Biology.

[b7] Bams RA (1976). Survival and propensity for homing as affected by presence or absence of locally adapted paternal genes in two transplanted populations of pink salmon (*Oncorhynchus* gorbuscha). Journal of the Fisheries Board of Canada.

[b8] Bond MH, Crane PA, Larson WA, Quinn TP (2014). Is isolation by adaptation driving genetic divergence among proximate Dolly Varden char populations?. Ecology and Evolution.

[b9] Bradbury IR, Hamilton LC, Robertson MJ, Bourgeois CE, Mansour A, Dempson JB, Marshall CT (2013). Landscape structure and climatic variation determine Atlantic salmon genetic connectivity in the Northwest Atlantic. Canadian Journal of Fisheries and Aquatic Sciences.

[b10] Broadhurst LM, Lowe A, Coates DJ, Cunningham AA, McDonald M, Vesk PA, Yates C (2008). Seed supply for broadscale restoration: maximizing evolutionary potential. Evolutionary Applications.

[b11] Browne J (1982). First Results from a New Method of Tagging Salmon – The Coded Wire Tag.

[b12] Byrne CJ, Poole R, Rogan G, Dillane M, Whelan KF (2003). Temporal and environmental influences on the variation in Atlantic salmon smolt migration in the Burrishoole system 1970–2000. Journal of Fish Biology.

[b13] Carlson SM, Satterthwaite WH, Fleming IA (2011). Weakened portfolio effect in a collapsed salmon population complex. Canadian Journal of Fisheries and Aquatic Sciences.

[b14] Crow JF, Morton NE (1955). Measurement of gene frequency drift in small populations. Evolution.

[b15] De Eyto E, McGinnity P, Huisman J, Coughlan J, Consuegra S, Farrell K, O'Toole C (2011). Varying disease-mediated selection at different life-history stages of Atlantic salmon in fresh water. Evolutionary Applications.

[b16] Dionne M, Caron F, Dodson JJ, Bernatchez L (2008). Landscape genetics and hierarchical genetic structure in Atlantic salmon: the interaction of gene flow and local adaptation. Molecular Ecology.

[b17] Donaghy MJ, Verspoor E (2000). A new design of instream incubator for planting out and monitoring Atlantic salmon eggs. North American Journal of Fisheries Management.

[b19] Einum S, Fleming IA (1999). Maternal effects of egg size in brown trout (*Salmo trutta*): norms of reaction to environmental quality. Proceedings of the Royal Society of London Series B: Biological Sciences.

[b20] Eliason EJ, Clark TD, Hague MJ, Hanson LM, Gallagher ZS, Jeffries KM, Gale MK (2011). Differences in thermal tolerance among sockeye salmon populations. Science.

[b21] Endler J, Mazer S, Williams M, Sandoval C, Ferren W (2010). http://nrs.ucop.edu/research/guidelines/non_native_genotypes.htm.

[b22] Fleming IA, Einum S (1997). Experimental tests of genetic divergence of farmed from wild Atlantic salmon due to domestication. ICES Journal of Marine Science: Journal du Conseil.

[b23] Fraser DJ, Bernatchez L (2001). Adaptive evolutionary conservation: towards a unified concept for defining conservation units. Molecular Ecology.

[b24] Fraser DJ, Cook AM, Eddington JD, Bentzen P, Hutchings JA (2008). Mixed evidence for reduced local adaptation in wild salmon resulting from interbreeding with escaped farmed salmon: complexities in hybrid fitness. Evolutionary Applications.

[b25] Fraser DJ, Weir LK, Bernatchez L, Hansen MM, Taylor EB (2011). Extent and scale of local adaptation in salmonid fishes: review and meta-analysis. Heredity.

[b26] Garant D, Dodson JJ, Bernatchez L (2003). Differential reproductive success and heritability of alternative reproductive tactics in wild Atlantic salmon (*Salmo salar* L.). Evolution.

[b27] Garcia de Leaniz C, Fleming IA, Einum S, Verspoor E, Jordan WC, Consuegra S, Aubin-Horth N (2007). A critical review of adaptive genetic variation in Atlantic salmon: implications for conservation. Biological Reviews.

[b28] Gilk SE, Wang IA, Hoover CL, Smoker WW, Taylor SG, Gray AK, Gharrett AJ (2004). Outbreeding depression in hybrids between spatially separated pink salmon, *Oncorhynchus gorbuscha*, populations: marine survival, homing ability, and variability in family size. Environmental Biology of Fishes.

[b29] Glover KA, Quintela M, Wennevik V, Besnier F, Sørvik AGE, Skaala Ø (2012). Three decades of farmed escapees in the wild: a spatio-temporal analysis of Atlantic salmon population genetic structure throughout Norway. PLoS One.

[b31] Green BS (2008). Maternal effects in fish populations. Advances in Marine Biology.

[b32] Greene CM, Hall JE, Guilbault KR, Quinn TP (2010). Improved viability of populations with diverse life-history portfolios. Biology Letters.

[b33] Hannon RM, Meek TH, Acosta W, Maciel RC, Schutz H, Garland T (2011). Sex-specific heterosis in line crosses of mice selectively bred for high locomotor activity. Behavior Genetics.

[b34] Hansen MM, Ruzzante DE, Nielsen EE, Bekkevold D, Mensberg K-LD (2002). Long-term effective population sizes, temporal stability of genetic composition and potential for local adaptation in anadromous brown trout (*Salmo trutta*) populations. Molecular Ecology.

[b35] Hansen LP, Holm M, Holst JC, Mills D, Jacobsen JA (2003). The ecology of post-smolts of Atlantic salmon. Salmon at the Edge.

[b36] Hatfield T, Schluter D (1999). Ecological speciation in sticklebacks: environment-dependent hybrid fitness. Evolution.

[b37] Heath DD, Fox CW, Heath JW (1999). Maternal effects on offspring size: variation through early development of chinook salmon. Evolution.

[b38] Hendry AP, Wenburg JK, Bentzen P, Volk EC, Quinn TP (2000). Rapid evolution of reproductive isolation in the wild: evidence from introduced salmon. Science.

[b40] Hilborn R, Quinn TP, Schindler DE, Rogers DE (2003). Biocomplexity and fisheries sustainability. Proceedings of the National Academy of Sciences.

[b41] Hindar K, Fleming IA, McGinnity P, Diserud O (2006). Genetic and ecological effects of salmon farming on wild salmon: modelling from experimental results. ICES Journal of Marine Science: Journal du Conseil.

[b42] Hines RO, Hines WGS, Robinson BW (2004). A new statistical test of fitness set data from reciprocal transplant experiments involving intermediate phenotypes. The American Naturalist.

[b43] Houde AL, Fraser DJ, O'Reilly P, Hutchings JA (2011). Relative risks of inbreeding and outbreeding depression in the wild in endangered salmon. Evolutionary Applications.

[b44] Hufford KM, Mazer SJ (2003). Plant ecotypes: genetic differentiation in the age of ecological restoration. Trends in Ecology and Evolution.

[b45] Hutchings JA, Fraser DJ (2008). The nature of fisheries- and farming-induced evolution. Molecular Ecology.

[b47] Jonsson B, Jonsson N (2009). A review of the likely effects of climate change on anadromous Atlantic salmon *Salmo salar* and brown trout *Salmo trutta*, with particular reference to water temperature and flow. Journal of Fish Biology.

[b48] Kallio-Nyberg I, Koljonen M-L, Saloniemi I (2000). Effect of maternal and paternal line on spatial and temporal marine distribution in Atlantic salmon. Animal Behaviour.

[b49] Kawecki TJ, Ebert D (2004). Conceptual issues in local adaptation. Ecology Letters.

[b1000] King TL, Eackles S, Letcher BH (2005). Microsatellite DNA markers for the study of Atlantic salmon (*Salmo salar*) kinship, population structure, and mixed-fishery analyses. Molecular Ecology Notes.

[b50] Larson WA, Seeb JE, Dann TH, Schindler DE, Seeb LW (2014). Signals of heterogeneous selection at an MHC locus in geographically proximate ecotypes of sockeye salmon. Molecular Ecology.

[b51] Lin J, Quinn TP, Hilborn R, Hauser L (2008). Fine-scale differentiation between sockeye salmon ecotypes and the effect of phenotype on straying. Heredity.

[b2000] Lynch M, Walsh B (1998). Genetics and Analysis of Quantitative Traits.

[b52] McGinnity P, Stone C, Taggart JB, Cooke D, Cotter D, Hynes R, McCamley C (1997). Genetic impact of escaped farmed Atlantic salmon (*Salmo salar* L.) on native populations: use of DNA profiling to assess freshwater performance of wild, farmed, and hybrid progeny in a natural river environment. ICES Journal of Marine Science: Journal du Conseil.

[b53] McGinnity P, Prodöhl P, Ferguson A, Hynes R, Ó Maoiléidigh N, Baker N, Cotter D (2003). Fitness reduction and potential extinction of wild populations of Atlantic salmon, *Salmo salar*, as a result of interactions with escaped farm salmon. Proceedings of the Royal Society of London Series B: Biological Sciences.

[b54] McGinnity P, Prodöhl P, Ó Maoiléidigh N, Hynes R, Cotter D, Baker N, O'Hea B (2004). Differential lifetime success and performance of native and non-native Atlantic salmon examined under communal natural conditions. Journal of Fish Biology.

[b55] McGinnity P, Jennings E, Allott N, Samuelsson P, Rogan G, Whelan K, Cross T (2009). Impact of naturally spawning captive-bred Atlantic salmon on wild populations: depressed recruitment and increased risk of climate-mediated extinction. Proceedings of the Royal Society of London B: Biological Sciences.

[b56] Moore J-S, Harris LN, Tallman RF, Taylor EB, Morán P (2013). The interplay between dispersal and gene flow in anadromous Arctic char (*Salvelinus alpinus*): implications for potential for local adaptation. Canadian Journal of Fisheries and Aquatic Sciences.

[b57] Moore JW, Yeakel JD, Peard D, Lough J, Beere M (2014). Life-history diversity and its importance to population stability and persistence of a migratory fish: steelhead in two large North American watersheds. Journal of Animal Ecology.

[b58] Myers JM, Heggelund PO, Hudson G, Iwamoto RN (2001). Genetics and broodstock management of coho salmon. Aquaculture.

[b3000] O'Reilly PT, Hamilton LC, McConnell SK, Wright JM (1996). Rapid analysis of genetic variation in Atlantic salmon (*Salmo salar*) by PCR multiplexing of dinucleotide and tetranucleotide microsatellites. Canadian Journal of Fisheries and Aquatic Sciences.

[b59] Ó Maoiléidigh N, Browne J, Cullen A, McDermott T, Keatinge M (1994).

[b4000] Paterson S, Piertney SB, Knox D, Gilbey J, Verspoor E (2004). Characterization and PCR multiplexing of novel highly variable tetranucleotide Atlantic salmon (*Salmo salar* L.) microsatellites. Molecular Ecology Notes.

[b60] Phillimore AB, Hadfield JD, Jones OR, Smithers RJ (2010). Differences in spawning date between populations of common frog reveal local adaptation. Proceedings of the National Academy of Sciences.

[b61] Pinheiro J, Bates D, DebRoy SS, Sarkar D (2013).

[b62] Pujolar JM, Vincenzi S, Zane L, Jesensek D, De Leo GA, Crivelli AJ (2011). The effect of recurrent floods on genetic composition of marble trout populations. PLoS One.

[b63] Quinn TP (2005). The Behavior and Ecology of Pacific Salmon and Trout.

[b5000] R Development Core Team (2008). R: A Language and Environment for Statistical Computing.

[b64] Raleigh RF (1971). Innate control of migrations of salmon and trout fry from natal gravels to rearing areas. Ecology.

[b66] Richardson JL, Urban MC, Bolnick DI, Skelly DK (2014). Microgeographic adaptation and the spatial scale of evolution. Trends in Ecology and Evolution.

[b67] Ricker WE, Simon RC, Larkin PA (1972). Hereditary and environmental factors affecting certain salmonid populations. The Stock Concept in Pacific Salmon.

[b68] Rogers LA, Schindler DE (2008). Asynchrony in population dynamics of sockeye salmon in southwest Alaska. Oikos.

[b69] Ruff CP, Schindler DE, Armstrong JB, Bentley KT, Brooks GT, Holtgrieve GW, McGlauflin MT (2011). Temperature-associated population diversity in salmon confers benefits to mobile consumers. Ecology.

[b70] Schindler DE, Hilborn R, Chasco B, Boatright CP, Quinn TP, Rogers LA, Webster MS (2010). Population diversity and the portfolio effect in an exploited species. Nature.

[b71] Schindler DE, Armstrong JB, Bentley KT, Jankowski K, Lisi PJ, Payne LX (2013). Riding the crimson tide: mobile terrestrial consumers track phenological variation in spawning of an anadromous fish. Biology Letters.

[b6000] Slettan A, Olsaker I, Lie Ø (1995). Atlantic salmon, *Salmo salar*, microsatellites at the SSOSL25, SSOSL85, SSOSL311, SSOSL417 loci. Animal Genetics.

[b72] Sokal RR, Rohlf FJ (1995). Biometry: The Principles and Practice of Statistics in Biological Research.

[b73] Tallmon DA, Luikart G, Waples RS (2004). The alluring simplicity and complex reality of genetic rescue. Trends in Ecology and Evolution.

[b7000] Taggart JB (2007). FAP: an exclusion-based parental assignment program with enhanced predictive functions. Molecular Ecology Notes.

[b74] Taylor EB (1991). A review of local adaptation in Salmonidac, with particular reference to Pacific and Atlantic salmon. Aquaculture.

[b75] Waples RS (1991). Pacific salmon, *Oncorhynchus* spp., and the definition of “species” under the Endangered Species Act. Marine Fisheries Review.

[b76] Weeks AR, Sgro CM, Young AG, Frankham R, Mitchell NJ, Miller KA, Byrne M (2011). Assessing the benefits and risks of translocations in changing environments: a genetic perspective. Evolutionary Applications.

[b78] Westley PA, Ward EJ, Fleming IA (2012). Fine-scale local adaptation in an invasive freshwater fish has evolved in contemporary time. Proceedings of the Royal Society of London B: Biological Sciences.

[b79] Whiteley AR, Fitzpatrick SW, Funk WC, Tallmon DA (2015). Genetic rescue to the rescue. Trends in Ecology & Evolution.

[b80] Wilkins NP, Cotter D, O'Maoiléidigh N (2001). Ocean migration and recaptures of tagged, triploid, mixed-sex and all-female Atlantic salmon (*Salmo salar* L.) released from rivers in Ireland. Genetica.

[b81] Zippin C (1958). The removal method of population estimation. The Journal of Wildlife Management.

[b82] Zuur A, Ieno EN, Walker N, Saveliev AA, Smith GM (2009). Mixed Effects Models and Extensions in Ecology with R.

