# Supplementary material for: The signature of fine scale local adaptation in Atlantic salmon revealed from common garden experiments in nature
Source: Evol Appl. 2015 Sep 11;8(9):881–900. doi: 10.1111/eva.12299 (PMC4610385; doi:10.1111/eva.12299)
Supplement: Supplementary file 1 — Figure S1. Progeny fork length and mass versus mean eyed-egg diameter and dam fork length, plotted separately for each life/sampling stage. [file eva0008-0881-sd1.pdf]

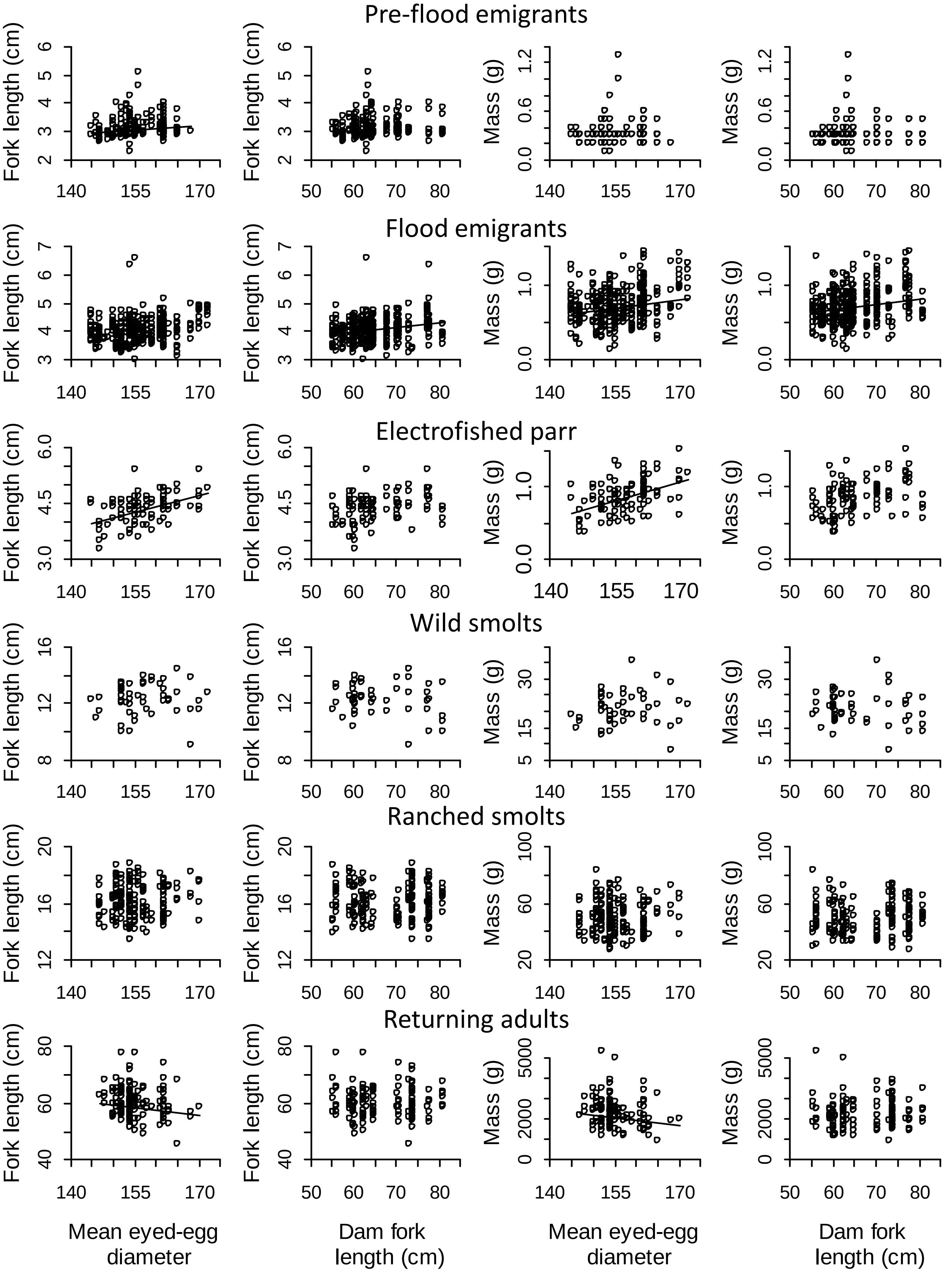

Pre-flood emigrants
Flood emigrants
Electrofished parr
Wild smolts
Ranched smolts
Returning adults
Fork length (cm)
Mass (g)
Mean eyed-egg diameter
Dam fork length (cm)

Fig. S1: Progeny fork length and mass versus mean eyed-egg diameter and dam fork length, plotted separately for each life/sampling stage. Top row of panels: pre-flood emigrants. Second row from top: flood emigrants. Third row from top: electrofished parr. Third row from bottom: wild smolts. Second row from bottom: hatchery-produced ranched smolts. Bottom row: returning adults. Lines show predicted effects from LMMs, where significant.
