# Supplementary material for: The signature of fine scale local adaptation in Atlantic salmon revealed from common garden experiments in nature
Source: Evol Appl. 2015 Sep 11;8(9):881–900. doi: 10.1111/eva.12299 (PMC4610385; doi:10.1111/eva.12299)
Supplement: Supplementary file 2 — Appendix S1. Broodstock details. [file eva0008-0881-sd2.pdf]

Appendix I: Broodstock details

Local (Burrishoole) Females

Sample Code	BF_1	BF_2	BF_3	BF_4	BF_5	BF_6	BF_7	BF_8	BF_9	BF_11	BF_12	BF_14	BF_13
Date Stripped	22/12/2008									29/12/2008			14/1/2009
Fork length (cm)	60.0	77.2	59.2	73.2	57.5	63.1	60.4	63.5	57.7	64.5	81.0	56.3	74.0
Sea age	1SW	2SW	1SW	2SW	1SW	1SW	1SW	2SW	1SW	2SW	2SW	1SW	2SW
TOTAL n(eggs) per hen	3352	3844	3123	3379	1047*	4026	2696	1738*	2474	3659	5053	3602	6931
Total n(eggs) stripped	3063	3610	2800	3033	970	3444	2569	1461	2333	3400	4472	3534	6714
n(eggs) crossed with Burrishoole male	1547	1807	1400	1533	508	1711	1325	722	1160	1800	2336	1867	3381
n(eggs) crossed with Owenmore male	1516	1803	1400	1500	462	1733	1244	739	1173	1600	2136	1667	3333
n(eggs) retained in cavity	289	234	323	346	77	582	127	277	141	259	581	68	217
Volume of 200 eyed eggs (mls)	47.5	61.0	45.0	56.5	39.0	45.0	46.5	46.0	37.5	45.0	44.0	42.0	42.0

Local (Burrishoole) Males

Sample code	BM_2	BM_3	BM_5	BM_4	BM_7	BM_8	BM_11	BM_12	BM_13	BM_14	BM_10
Date stripped*	22/12 and 29/12/08			22/12/2008						29/12	14/01/2009
Fork length (cm)	60.4	61.0	63.4	60.8	62.5	55.8	60.3	54.8	52.8	61.5	62.5
Sea age	1SW	1SW	1SW	1SW	1SW	1SW	1SW	1SW	1SW	1SW	1SW

Foreign (Owenmore) Females

Sample code	OF_1	OF_2	OF_3	OF_4	OF_5	OF_6	OF_7	OF_8	OF_9	OF_10	OF_11	OF_12	OF_13
Date stripped	22/12/2008								29/12/2008				14/01/2009
Fork length (cm)	70.2	64.5	62.4	70.5	68	64.5	77.8	60.4	61	64.9	62	60	64.5
Age	2SW	1SW	1SW	2SW	2SW	1SW	2SW	1SW	1SW	1SW	1SW	1SW	1SW
Total n(eggs) per hen	4096	4634	4270	4134	3443	3829	7650	3905	3279	4644	3047	3796	4173
Total n(eggs) stripped	3904	4377	4155	3719	3254	3571	7386	3700	3078	4427	2879	3621	4133
n(eggs) crossed with Owenmore male	1904	2333	2064	1756	1618	1863	3719	1791	1615	2291	1521	1884	2089
n(eggs) crossed with Burrishoole male	2000	2044	2091	1963	1636	1708	3667	1909	1463	2136	1358	1737	2044
n(eggs) retained in cavity	192	257	115	415	189	258	264	205	201	217	168	175	40
volume of 200eggs (mls)	54.0	45.0	44.0	54.0	55.0	44.5	54.0	44.0	41.0	44.0	53.0	38.0	45.0

Foreign (Owenmore) Males

Sample code	OM_1	OM_2	OM_3	OM_4	OM_5	OM_6	OM_7	OM_8	OM_9	OM_10	OM_11	OM_12	OM_13	OM_14
Date stripped	22/12/2008									29/12/2008				14/01/2009
Fork length (cm)	64.6	68.5	64.0	73.1	58.0	68.2	66.8	61.0	67.0	74.0	84.5	65.1	62.2	76.0
Age	1SW	1SW	1SW	2SW	1SW	1SW	1SW	1SW	1SW	2SW	2SW	1SW	1SW	2SW

* These Local females had already shed a fraction of their eggs prior to stripping and hence fewer eggs were stripped; these females are excluded when comparing fecundities (see below).

Statistical tests for body size, egg size and fecundity differences among broodstock groups:

ANOVA analysis showed that Local and Foreign dams did not differ significantly in fork-length L_F ($F_{1,23}$ = 0.37, P = 0.55), controlling for the fact that 2SW dams were bigger than 1SW dams ($F_{1,23}$ = 56.4, P < 0.001). The L_F difference between 2SW and 1SW dams was not significantly different for Local versus Foreign fish (origin river × sea age interaction: $F_{1,23}$ = 4.05, P = 0.06). Eyed egg volume was larger for 2SW dams ($F_{1,23}$ = 18.54, P < 0.001) but no different for Local versus Foreign dams ($F_{1,23}$ = 0.61, P = 0.44), nor was there a significant interaction between origin river and sea age ($F_{1,23}$ = 0.67, P = 0.42). Foreign females in this sample did not produce any more eggs per kg of body mass compared to Local females ($F_{1,21}$ = 0.15, P = 0.70), controlling for the fact that 1SW dams produced slightly more (~398) eggs per kg of body mass than 2SW dams ($F_{1,21}$ = 5.11, P = 0.03). Overall fecundity (total number of eggs per hen) was not different between Local and Foreign dams ($F_{1,21}$ = 0.74, P = 0.40) and that between 1SW and 2SW dams was marginally non-significant ($F_{1,22}$ = 3.46, P = 0.08). Foreign 1SW sires were significantly larger than Local 1SW sires ($F_{1,19}$ =10.73, P = 0.004) and Foreign 2SW sires were significantly larger than Foreign 1SW sires ($F_{1,12}$ =28.61, P < 0.001). There were no 2SW Local sires.

Mating scheme and eyed-eggs planted to river (or retained in hatchery for ranching) per full-sib family: The two families produced by OF_13 exhibited anomalously low alevin survival in the hatchery and were excluded in the calculations of expected ranched smolts per group.

	BF_1	BF_11	BF_12	BF_13	BF_14	BF_2	BF_3	BF_4	BF_5	BF_6	BF_7	BF_8	BF_9	OF_1	OF_10	OF_11	OF_12	OF_13	OF_2	OF_3	OF_4	OF_5	OF_6	OF_7	OF_8	OF_9
BM_10				1285 (1047)														1260 (0)								
BM_11										1302 (321)													1000 (0)			
BM_12												631 (0)										1019 (0)				
BM_13													1089 (0)	1037 (528)												
BM_14					1250 (166)										1285 (675)											
BM_2			1272 (722)					1054 (102)																	1000 (0)	710 (0)
BM_3		1333 (314)							451 (0)							926 (130)				1000 (571)						
BM_4						1066 (0)													1023 (0)							
BM_5	1276 (166)																1000 (285)									
BM_7							1022 (204)																	1020 (510)		
BM_8											609 (174)										1000 (520)					
OM_1									380 (0)														1000 (0)			
OM_10		1255 (1162)														975 (0)										
OM_11			1304 (129)																							896 (44)
OM_12																	974 (0)									
OM_13					1250 (152)										1255 (190)											
OM_14				1285 (880)														1000 (0)								
OM_2													1027 (0)									1035 (0)				
OM_3							720 (0)														1020 (0)					
OM_4												609 (0)								1000 (524)						
OM_5											826 (0)								1023 (0)							
OM_6	1020 (285)																							1020 (1019)		
OM_7								1018 (120)						1056 (592)												
OM_8										1318 (309)															1000 (409)	
OM_9						1300 (306)																				
