# Supplementary material for: The signature of fine scale local adaptation in Atlantic salmon revealed from common garden experiments in nature
Source: Evol Appl. 2015 Sep 11;8(9):881–900. doi: 10.1111/eva.12299 (PMC4610385; doi:10.1111/eva.12299)
Supplement: Supplementary file 3 — Appendix S2. Details of genomic DNA extraction, microsatellite PCR amplification and allele genotyping. [file eva0008-0881-sd3.pdf]

Appendix II

Genomic DNA was extracted from 994 individuals representing the freshwater phase of the study (wild parr and wild smolts) using the Qiagen kit following manufacturer's recommendations. The concentration of resulting genomic DNA was measured using a Nanodrop ND-1000 while quality (i.e. proportion of high molecular weight DBA) was assessed by running a subsample from each group of 96 individual samples on 1.5% 0.5X TBE pH 8.0 agarose gels stained with ethidium bromide. DNA was subsequently diluted to a working concentration ranging from 20 and 40 ng/µl for PCR amplification.

Microsatellite PCR amplifications were carried out in 10µl volumes. PCR for five of the microsatellite markers involved two multiplexes (primer concentrations given in Table 1). For the remaining three microsatellite markers PCR were carried out individually with primer concentration at 1µM. In addition to the PCR primers, each reaction contained 1µL of DNA (~20 - 40 ng), 0.25 mM dNTPs, 0.5 U *Taq* DNA polymerase and 2µl of 5x reaction buffer supplemented with 0.5mM $MgCl_2$. Cycling conditions followed an initial denaturation period of 95°C for 3 minutes, 30 cycles of 95°C for 30 seconds, 56°C for 30 seconds and 72°C for 30 seconds, followed by a final extension step at 72°C for 5 minutes. Microsatellite alleles were resolved using 6% denatured polyacrylamide gels on a LI-COR 4300 DNA analyser. The sizes and weights of alleles were determined using a molecular weight marker (LI-COR) and a customised allele cocktail made of a mixture of the most common genotypes for these markers loci determined as part of previous studies within our research group to ensure consistent scoring.

Table 1. Final microsatellite primer concentration in multiplex reaction for markers screened in the LI-COR 4300 DNA analyser.

	**Loci**	**Primer concentration (µM) in PCR**
**Multiplex 1**	197	0.5
	171	2.0
	3016	2.0
**Multiplex 2**	*SsaD71*	2.0
	Sssp2216	0.25

The remaining samples (returning adults) were analysed for the same loci on an ABI 3730XL 96 capillary DNA analyser (Life Technologies). For these samples, genomic DNA was extracted from biopsy tissue samples using the Wizard SV96 Genomic DNA Purification System (Promega Ltd) according to manufactures instructions. Assessment of DNA concentration and quality was carried out as described earlier for samples screened for the LICOR system. The main difference was that for samples screened on the ABI 3730XL DNA analyser, DNA was diluted to a working concentration ranging between 4 and 8 ng/µl for PCR amplifications, which were carried out in 3.5µl volumes. PCR primer concentrations and information whether markers were screened as part of multiplexes or individually are given in Table 2. Each PCR reaction included 1µl DNA (4 -8 ng), primers, and 1.75µl of Plain Combi PP Master Mix (Top-Bio). Cycling conditions were as follows; an initial denaturation period of 95°C for 15 minutes, 5 cycles of 94°C for 30 seconds, 55°C for 90 seconds and 72°C for 60 seconds, 22 cycles of 94°C for 30 seconds, 57°C for 90 seconds and

72°C for 60 seconds, followed by a final extension at 60°C for 30 minutes. Fragment length was determined using GeneScan 600-LIZ size standard and alleles were scored using Genemarker v1.6 (Applied Biosystems).

Table 2. Final microsatellite primer concentration in multiplex reaction for markers screened in the ABI 3730XL DNAA analyser.

	Loci	Primer concentration (µM) in PCR
**Single PCR**	***Ssa197***	0.02
**Single PCR**	***SsaD71***	0.08
**Single PCR**	***Sssp3016***	0.04
**Multiplex 1**	***Sssp2210***	0.03
	SsaD170	0.06
	Ssa171	0.06
**Multiplex 2**	***Sssp2216***	0.02
	SSOSL85	0.12

Allele calls from both DNA analysers were calibrated using control samples of known genotypes. Raw alleles calls were standardised by correcting alleles from the ABI system to match those sizes recorded for genotypes analysed using the LI-COR system.
