# Supplementary material for: The signature of fine scale local adaptation in Atlantic salmon revealed from common garden experiments in nature
Source: Evol Appl. 2015 Sep 11;8(9):881–900. doi: 10.1111/eva.12299 (PMC4610385; doi:10.1111/eva.12299)
Supplement: Supplementary file 4 — Appendix S3. Results of the representation GLMMs. [file eva0008-0881-sd4.pdf]

Appendix III: Results of the representation GLMMs

Results of the generalised linear mixed-models comparing progeny representation among groups and with respect to other explanatory variables. Group was a factor with four levels: $\text{Local}_{\text{female}}$ x $\text{Local}_{\text{male}}$, $\text{Local}_{\text{female}}$ x $\text{Foreign}_{\text{male}}$, $\text{Foreign}_{\text{female}}$ x $\text{Local}_{\text{male}}$ and $\text{Foreign}_{\text{female}}$ x $\text{Foreign}_{\text{male}}$. Date planted was a factor with three levels: early, mid and late. Dam and sire life-history were each two level factors (1SW versus 2SW). Results are presented as an ANOVA table based on Type II tests, with non-significant terms removed sequentially until all remaining terms were significant at the 5% level. Random effects of dam and sire were included in all models (*Dam* = variance attributable to differences among dams; *Sire* = variance attributable to sires nested within dams; *Res* = residual variance). *k* = dispersion parameter from the negative binomial (where the variance = $\mu + \mu^2/k$). All estimates are on log scale. Models were fitted using the glmmPQL function in the MASS library in R (Venables and Ripley 2002).

Life stage	Fixed effect	*NumDF*	*DenDF*	*F*	*P*	*k*	*Dam*	*Sire*	*Res*
Pre-flood dispersal	Intercept	1	23	371.00	<0.001	1.28	0.57	0.71	<0.01
	Group	3	23	3.61	0.029				
	Date planted	2	23	831.73	<0.001				
	Non-significant terms								
	Dam L_F	1	21	1.57	0.224				
	Dam life-history	1	22	1.81	0.192				
	Sire life-history	1	21	2.48	0.131				
	Eyed-egg diameter	1	22	0.31	0.581				
Flood migration	Intercept	1	26	3131	<0.001	13.29	0.02	0.03	0.76
	Date planted	2	23	12.74	<0.001				
	Non-significant terms								
	Group	3	23	1.81	0.173				
	Dam L_F	1	21	1.66	0.212				
	Dam life-history	1	22	1.02	0.324				
	Sire life-history	1	21	0.38	0.543				
	Eyed-egg diameter	1	22	1.02	0.323				
Electro-fished parr	Intercept	1	26	2408	<0.001	20.8	<0.01	<0.01	1.04
	Date planted	2	23	3.86	0.036				
	Non-significant terms								
	Group	3	23	1.05	0.390				
	Dam L_F	1	22	1.25	0.276				
	Dam life-history	1	21	0.29	0.600				
	Sire life-history	1	21	0.29	0.600				
	Eyed-egg diameter	1	22	0.47	0.502				
Presmolts + smolts	Intercept	1	26	723	<0.001	5.30	0.59	1.36	<0.01
	Date planted	2	23	487.30	<0.001				
	Non-significant terms								
	Group	3	22	1.56	0.228				
	Dam L_F	1	22	0.51	0.481				
	Dam life-history	1	21	2.60	0.122				
	Sire life-history	1	21	1.59	0.221				
	Eyed-egg diameter	1	25	1.81	0.191				
Adult returns	Intercept	1	19	253.5	<0.001	5.35	0.08	0.31	0.21
	Group	3	9	15.90	<0.001				
	Non-significant terms								
	Dam L_F	1	18	0.51	0.483				
	Dam life-history	1	17	<0.001	0.985				
	Sire life-history	1	7	0.89	0.376				

	Eyed-egg diameter	1	7	0.37	0.560

References:

Venables, W. N. & Ripley, B. D. (2002) Modern Applied Statistics with S. Fourth Edition. Springer, New York.
