# Supplementary material for: The signature of fine scale local adaptation in Atlantic salmon revealed from common garden experiments in nature
Source: Evol Appl. 2015 Sep 11;8(9):881–900. doi: 10.1111/eva.12299 (PMC4610385; doi:10.1111/eva.12299)
Supplement: Supplementary file 5 — Appendix S4. Estimating lifetime success of each group [file eva0008-0881-sd5.pdf]

Appendix IV: Estimating lifetime success of each group

Estimates of the lifetime success of each group, defined as the average number of green-eggs produced (i.e. at the beginning of the next generation) per eyed-egg planted out, were calculated as follows (see also Table 3 in main text). First, fecundity $F_{i,j}$ (expected number of green-eggs per returning adult female *i* in group *j*) was estimated as $M_{i,j}\hat{f}$, where $M_{i,j}$ was the mass (kg) of each returning female and $\hat{f}$ is our estimate of mass-specific fecundity (Local females on average produce approximately 1400 eggs/kg, while Foreign females produce approximately 1600 eggs/kg, so $\hat{f}$ was set to 1500). Next, an estimate of the total number of green-eggs produced by the N_j returning adult females in each group was calculated as $\sum_{i=1}^{N_j} F_{i,j}$. These were then converted to expected numbers of green-eggs per female smolt that survives to become an adult by dividing by half the estimated number of ranched smolts per group (based on initial egg numbers, equal egg-to-smolt survival rates in the hatchery and an assumed 50:50 sex ratio). The previous two steps thus accounted for group differences in smolt-to-adult survival, as estimated based on the ranched smolts. The expected numbers of green-eggs per female smolt (that survives to become an adult) per group were then multiplied by half the actual numbers of wild smolts (again assuming an equal sex ratio) produced per experimental group, giving an estimate of the total number of eggs produced by wild smolts in each group. This was then divided by the total number of eyed-eggs planted out per group, to arrive at estimates of the mean absolute lifetime success of each group. Finally, the absolute lifetime success of each group was divided by the lifetime success of the $\text{Local}_{\text{female}}$ x $\text{Local}_{\text{male}}$ group to obtain estimates of lifetime success relative to 'pure natives'. Suitable habitat for juvenile salmonids is present in the home river downstream of the experiment-trap and in freshwater Lough Feeagh. Thus, parr emigrating from the experiment-river would potentially be able to survive and produce smolts. A second measure of wild smolt output was calculated by assuming that emigrant parr had the same survival

downstream as parr of the equivalent group remaining in the experiment-river. This second measure combines the estimated number of smolts produced from these emigrants with the actual experiment-trap smolts, to give an estimate of total sea-entry smolts per group. The relative lifetime success of each group was then recalculated based on this second measure of wild smolt output.
