# Supplementary material for: The signature of fine scale local adaptation in Atlantic salmon revealed from common garden experiments in nature
Source: Evol Appl. 2015 Sep 11;8(9):881–900. doi: 10.1111/eva.12299 (PMC4610385; doi:10.1111/eva.12299)
Supplement: Supplementary file 6 — Appendix S5. Survival of subsampled eyed eggs retained in the hatchery from all families outplanted in the experimental river. [file eva0008-0881-sd6.pdf]

Appendix V: Survival of subsampled eyed eggs retained in the hatchery from all families outplanted in the experimental river.

Table 1: Survival of subsampled eyed eggs retained in the hatchery from all families outplanted in the experimental river.

Vibert Box No.	Stripping Date	Female Parent	Male Parent	Starting n(eggs)	n(morts) eggs	Date all hatched	n(morts) alveins	n(morts) total	n(with deformities)
**1A**	22/12/2008	**OF_6**	**OM_1**	25	0	18/03/2009	0	0	0
**1B**	22/12/2008	**OF_5**	**OM_2**	25	1	20/03/2009	0	1	0
**2A**	22/12/2008	**OF_4**	**OM_3**	25	0	20/03/2009	0	0	0
**2B**	22/12/2008	**OF_3**	**OM_4**	25	0	20/03/2009	0	0	0
**3A**	22/12/2008	**OF_2**	**OM_5**	25	0	20/03/2009	0	0	0
**3B**	22/12/2008	**OF_7**	**OM_6**	25	1	20/03/2009	1	2	0
**4A**	22/12/2008	**OF_1**	**OM_7**	25	1	20/03/2009	0	1	0
**4B**	22/12/2008	**OF_8**	**OM_8**	25	0	20/03/2009	0	0	0
**5A**	22/12/2008	**OF_8**	**BM_2**	25	0	20/03/2009	0	0	0
**5B**	22/12/2008	**OF_3**	**BM_3**	25	0	20/03/2009	0	0	0
**6A**	22/12/2008	**OF_2**	**BM_4**	25	0	23/03/2009	0	0	0
**6B**	22/12/2008	**OF_7**	**BM_7**	25	0	23/03/2009	0	0	0
**7A**	22/12/2008	**OF_4**	**BM_8**	25	0	23/03/2009	0	0	0
**7B**	22/12/2008	**OF_6**	**BM_11**	25	0	23/03/2009	0	0	0
**8A**	22/12/2008	**OF_5**	**BM_12**	25	0	20/03/2009	0	0	0
**8B**	22/12/2008	**OF_1**	**BM_13**	25	0	20/03/2009	0	0	0
**9A**	22/12/2008	**BF_4**	**BM_2**	25	0	18/03/2009	0	0	0
**9B**	22/12/2008	**BF_5**	**BM_3**	25	3	20/03/2009	0	3	0

Table 1 continued

Vibert Box No.	Stripping Date	Female Parent	Male Parent	Starting n(fry)	n(morts) eggs	Date all hatched	n(morts) alveins	n(morts) total	n(with deformities)
**10A**	22/12/2008	**BF_2**	**BM_4**	25	0	20/03/2009	0	0	0
**10B**	22/12/2008	**BF_1**	**BM_5**	25	0	20/03/2009	0	0	0
**11A**	22/12/2008	**BF_3**	**BM_7**	25	5	20/03/2009	0	5	0
**11B**	22/12/2008	**BF_7**	**BM_8**	25	0	20/03/2009	0	0	0
**12A**	22/12/2008	**BF_6**	**BM_11**	25	7	20/03/2009	0	7	0
**12B**	22/12/2008	**BF_8**	**BM_12**	25	0	20/03/2009	0	0	0
**13A**	22/12/2008	**BF_9**	**BM_13**	25	0	23/03/2009	0	0	0
**13B**	22/12/2008	**BF_5**	**OM_1**	25	0	18/03/2009	0	0	1 curved spine
**14A**	22/12/2008	**BF_9**	**OM_2**	25	0	23/03/2009	0	0	1 curved spine
**14B**	22/12/2008	**BF_3**	**OM_3**	25	0	20/03/2009	0	0	0
**15A**	22/12/2008	**BF_8**	**OM_4**	25	1	20/03/2009	0	1	0
**15B**	22/12/2008	**BF_7**	**OM_5**	25	0	20/03/2009	0	0	0
**16A**	22/12/2008	**BF_1**	**OM_6**	25	0	20/03/2009	0	0	0
**16B**	22/12/2008	**BF_4**	**OM_7**	25	0	20/03/2009	1	1	0
**17A**	22/12/2008	**BF_6**	**OM_8**	25	1	18/03/2009	0	1	0
**17B**	22/12/2008	**BF_2**	**OM_9**	25	0	20/03/2009	0	0	0
**18A**	29/12/2008	**OF_11**	**OM_10**	25	0	25/03/2009	0	0	0
**18B**	29/12/2008	**OF_9**	**OM_11**	25	0	27/03/2009	0	0	0
**19A**	29/12/2008	**OF_12**	**OM_12**	25	0	25/03/2009	0	0	0
**19B**	29/12/2008	**OF_10**	**OM_13**	25	0	25/03/2009	0	0	0
**20A**	29/12/2008	**OF_9**	**BM_2**	25	0	25/03/2009	0	0	0
**20B**	29/12/2008	**OF_10**	**BM_14**	25	0	25/03/2009	0	0	0
**21A**	29/12/2008	**OF_11**	**BM_3**	25	0	25/03/2009	0	0	0
**21B**	29/12/2008	**OF_12**	**BM_5**	25	0	25/03/2009	0	0	0
**22A**	29/12/2008	**BF_12**	**BM_2**	25	0	27/03/2009	0	0	1 curved spine
**22B**	29/12/2008	**BF_14**	**BM_14**	25	0	27/03/2009	1	1	0
**23A**	29/12/2008	**BF_11**	**BM_3**	25	2	27/03/2009	1	3	0
**23B**	29/12/2008	**BF_11**	**OM_10**	25	5	25/03/2009	0	5	0
**24A**	29/12/2008	**BF_12**	**OM_11**	25	5	27/03/2009	0	5	0
**24B**	29/12/2008	**BF_14**	**OM_13**	25	0	27/03/2009	0	0	0
**25A**	14/01/2009	**BF_13**	**BM_10**	25	0	22/04/2009	0	0	0
**25B**	14/01/2009	**BF_13**	**OM_14**	25	0	22/04/2009	0	0	0
**26A**	14/01/2009	**OF_13**	**OM_14**	25	0	22/04/2009	11	11	0
**26B**	14/01/2009	**OF_13**	**BM_10**	25	0	22/04/2009	24	24	0

Statistical analysis of survival from fertilisation to eyed-egg stage in the hatchery

A binomial GLMM (fit using penalised quasi-likelihood with the *glmmPQL* function in the R library *nlme*) including dam and sire as random effects was used to analyse variation in survival (at the full-sib family level) from fertilisation to eyed-egg stage in the hatchery. The response variable was a concatenated vector of the number of surviving eggs and the number non-surviving; all estimates and variance components are on the logit scale. Number of observations: 52; n= 26 dams; n = 25 sires. Group was a factor with four levels: $\text{Local}_{\text{female}}$ x $\text{Local}_{\text{male}}$, $\text{Local}_{\text{female}}$ x $\text{Foreign}_{\text{male}}$, $\text{Foreign}_{\text{female}}$ x $\text{Local}_{\text{male}}$ and $\text{Foreign}_{\text{female}}$ x $\text{Foreign}_{\text{male}}$. Stripping date was a factor with three levels: early, mid and late. Results are presented as an ANOVA table based on Type II tests, with non-significant terms removed sequentially until all remaining terms were significant at the 5% level.

Random effects:	*Variance*			
Dam	0.58			
Sire	0.33			
Residual	4.21			
Fixed effects:	NumDF	*DenDF*	*F*	*P*
Intercept	1	26	124.0	<0.001
Non-significant terms:				
Group	3	23	2.90	0.057
Stripping date	2	21	0.27	0.763
Dam L_F	1	20	0.06	0.811
Dam life-history	1	23	0.84	0.368
Sire life-history	1	22	4.10	0.055
Eyed-egg diameter	1	24	1.01	0.324
